# Coil combination for receive array spectroscopy: Are data‐driven methods superior to methods using computed field maps?

**DOI:** 10.1002/mrm.25618

**Published:** 2015-03-28

**Authors:** Christopher T. Rodgers, Matthew D. Robson

**Affiliations:** ^1^ Oxford Centre for Clinical Magnetic Resonance ResearchUniversity of Oxford, John Radcliffe HospitalOxfordUnited Kingdom

**Keywords:** MR spectroscopy, array, coil combination, WSVD, WSVD+Apod, WSVD+Apod+Blur, adaptive combination theory

## Abstract

**Purpose:**

Combining spectra from receive arrays, particularly X‐nuclear spectra with low signal‐to‐noise ratios (SNRs), is challenging. We test whether data‐driven combination methods are better than using computed coil sensitivities.

**Theory:**

Several combination algorithms are recast into the notation of Roemer's classic formula, showing that they differ primarily in their estimation of coil receive sensitivities. This viewpoint reveals two extensions of the whitened singular‐value decomposition (WSVD) algorithm, using temporal or temporal + spatial apodization to improve the coil sensitivities, and thus the combined spectral SNR.

**Methods:**

Radiofrequency fields from an array were simulated and used to make synthetic spectra. These were combined with 10 algorithms. The combined spectra were then assessed in terms of their SNR. Validation used phantoms and cardiac ^31^P spectra from five subjects at 3T.

**Results:**

Combined spectral SNRs from simulations, phantoms, and humans showed the same trends. In phantoms, the combined SNR using computed coil sensitivities was lower than with WSVD combination whenever the WSVD SNR was >14 (or >11 with temporal apodization, or >9 with temporal + spatial apodization). These new apodized WSVD methods gave higher SNRs than other data‐driven methods.

**Conclusion:**

In the human torso, at frequencies ≥49 MHz, data‐driven combination is preferable to using computed coil sensitivities. Magn Reson, 2015. © 2015 The Authors. Magnetic Resonance in Medicine published by Wiley Periodicals, Inc. on behalf of International Society for Magnetic Resonance in Medicine. This is an open access article under the terms of the Creative Commons Attribution License, which permits use, distribution and reproduction in any medium, provided the original work is properly cited. Magn Reson Med 75:473–487, 2016. © 2015 The Authors. Magnetic Resonance in Medicine published by Wiley Periodicals, Inc. on behalf of International Society for Magnetic Resonance in Medicine.

## INTRODUCTION

Magnetic resonance spectroscopy (MRS) opens a window on biochemical processes in healthy and diseased subjects 1. Most metabolites are present in small concentrations, so MRS methods and applications benefit particularly from an increased signal‐to‐noise ratio (SNR). Non‐proton spectroscopy, such as ^31^P‐MRS or ^13^C‐MRS, has even lower intrinsic SNR than ^1^H‐MRS because of the smaller gyromagnetic ratios of these nuclei. Thus, these methods are hindered even more acutely by their low SNR 2, 3.

In ^1^H‐MRS and MRI, receive arrays have largely superseded single‐element receive coils because receive arrays offer higher SNRs over larger fields of view in the same scan duration, and they enable parallel imaging. The theory of receive arrays 4, 5 applies to any nucleus, so X‐nuclear receive arrays ought to afford similar advantages. The problem of how best to combine the signals from a ^1^H receive array for imaging has been studied extensively 6, 7, 8. However, combining MR spectra is difficult because the single‐element spectra in each voxel contain important phase and frequency information that is not present in images 9. Combining X‐nuclear spectra is especially challenging because these spectra usually have low SNR and lack a strong reference signal such as the ^1^H water peak.

When an array's receive fields $B_{1}^{-}$ and noise covariance $\overset{⁁}{\Psi }$ are known, Roemer proved how to combine the signals to maximize SNR 4. Several authors have used the Biot‐Savart law or phantom replacement to estimate $B_{1}^{\pm }$ for coil combination, saturation correction, or sensitivity correction 10, 11, 12. Practically, these approaches are awkward for coil combination in vivo 10. It is not yet clear whether using computed $B_{1}^{-}$s yields a coil combination that is better or worse in vivo than that obtained by using a simpler data‐driven approach. We aim to answer this question in this study.

Specifically, we consider the following methods (detailed later). Each method computes a linear combination of the single‐element spectra for each voxel; yet each method obtains the weighting coefficients by different means.

1. “Roemer (BS B_1_^−^) combination”: Uses Roemer's formula (Eq. (4)) and Biot‐Savart law $B_{1}^{-}$s for each element to compute the maximum SNR combined spectrum.
2. “Roemer (BS B_1_^−^ phased) combination”: As in the above‐mentioned Roemer (BS B_1_^−^) combination method (#1), but scaling the Biot‐Savart law $B_{1}^{-}$s by a complex number per element.
3. “Roemer (Exact B_1_^−^) combination”: Uses Roemer's formula (Eq. (4)), with the exact $B_{1}^{-}$s (to give a best‐case result in simulations).
4. “Roemer (Phantom B_1_^−^) combination”: Uses Roemer's formula (Eq. (4)), with $B_{1}^{-}$s derived from phantom data.
5. “WSVD combination”: The authors' whitened singular value decomposition (WSVD) combination algorithm 9 uses a noise covariance prescan to decorrelate the single‐element spectra and then employs the singular value decomposition (SVD) to compute the maximum likelihood combined spectrum.
6. “WSVD+Apod combination”: A novel variant of the WSVD combination algorithm, introduced below, where we apply temporal apodization in a first step to better estimate the coil sensitivities before combining the original (i.e. not apodized) single‐element spectra.
7. “WSVD+Apod+Blur combination”: A second novel variant of the WSVD combination algorithm, also introduced below, where we apply temporal and spatial apodization in a first step to better estimate the coil sensitivities before combining the original (i.e. not apodized) single‐element spectra.
8. “Brown combination”: Brown's combination algorithm 13 uses the conjugate of the first point in each single‐element free‐induction decay (FID) as the combination weights.
9. “RefPeak combination”: Hall et al.'s algorithm 14 fits the single‐element spectra and then uses the complex amplitude of a reference peak to determine the combination weights.
10. “Generalized least squares (GLS) combination”: An et al.'s GLS algorithm 15 uses the complex integral over a reference peak to determine the coil sensitivities in Roemer's formula.

We begin by showing how all these algorithms relate to Roemer's classic maximum SNR combination formula. We explore the links to widely used MR image combination algorithms. We also discover how to improve on the WSVD combination algorithm by using additional prior knowledge. Then we compare the performance of these spectroscopy coil combination methods in simulations, phantoms, and a cardiac ^31^P‐MRS study in order to discover if data‐driven coil combination is better than using computed coil sensitivities.

## THEORY

### Signal Model

The radiofrequency (RF) field produced by a coil at **r** is $\text{Re}[{\overset{⁁}{B}}_{1}(r)\times e^{i\omega_{L}t}]$, where ${\overset{⁁}{B}}_{1}$ is a complex‐valued vector (denoted by a circumflex) and $\omega_{L}$ is the Larmor frequency. With the static field $B_{0}$ along the positive *z*‐axis, nuclear spins are excited by the clockwise component of the RF field:

(1) $${\overset{⁁}{B}}_{1}^{+}(\text{r)}=\text{(}{\overset{⁁}{B}}_{1x}(r)+i{\overset{⁁}{B}}_{1y}(r))/2\text{.}$$

The principle of reciprocity 16, 17, 18 shows that transverse magnetization ${\overset{⁁}{M}}_{xy}(r_{k})$, then induces a voltage

(2) $$s\propto {\left({\overset{⁁}{B}}_{1}^{-}\right)}^{*}{\overset{⁁}{M}}_{xy}(r_{k})+\text{noise}$$

in each element of the receive array where

(3) $${\left({\overset{⁁}{B}}_{1}^{-}\right)}^{*}=\text{(}{\overset{⁁}{B}}_{1x}(r)-i{\overset{⁁}{B}}_{1y}(r))/2\text{.}$$

Here, ${\overset{⁁}{B}}_{1}$ is the magnetic field that would arise hypothetically if this element were driven for *transmission* with a 1A current 4, 17, 19, 20, 21, 22, 23. In ultra‐high field imaging 24, the phases of the complex quantities ${\overset{⁁}{B}}_{1x}(r)$ and ${\overset{⁁}{B}}_{1y}(r)$ cause clearly visible features, which have allowed an unambiguous assignment of the phases in Equations (1), (2), (3) 16, 25, 26.

### The Roemer Combination Algorithm

Roemer 4 proved that a certain weighted sum of the single‐element spectra (or FIDs) from a receive array maximizes the SNR of the combined signal (for a point source or for a small, well localized voxel). This combination is unique apart from an overall arbitrary scaling and phase. Extending Roemer's notation (Eq. 32 in 4) for spectroscopy, the Roemer combined spectrum is:

(4) $$\overset{⁁}{P}(r_{k},\delta_{l})=C\frac{\overset{⁁}{p}{\left(r_{k},\delta_{l}\right)}^{T}{\left({\overset{⁁}{R}}^{-1}\right)}^{*}\overset{⁁}{b}(r_{k})}{\sqrt{\overset{⁁}{b}{\left(r_{k}\right)}^{†}{\left({\overset{⁁}{R}}^{-1}\right)}^{*}\overset{⁁}{b}(r_{k})}}.$$

where $\overset{⁁}{P}$ is a complex‐valued point in the combined spectrum at voxel $r_{k}$ with chemical shift $\delta_{l}$, $\overset{⁁}{p}$ is the column vector of points from the $N_{i}$ single‐element spectra, $\overset{⁁}{R}$ is the noise resistance matrix, $\overset{⁁}{b}(r_{k})$ is a column vector of $N_{i}$ single‐element ${\overset{⁁}{B}}_{1}^{-}$, C is an arbitrary complex scaling/phase constant, *^T^* denotes the transpose, * denotes the conjugate, and † denotes the conjugate transpose. Note that we have changed the location of the complex conjugates compared to Eq. 32 in 4 because Roemer assumed the noise resistance matrix $\overset{⁁}{R}$ to be real and symmetric, whereas it is actually complex and Hermitian (see Supporting Information).

To apply Equation (4), we must know $\overset{⁁}{R}$ and $\overset{⁁}{b}(r_{k})$. Now, the noise resistance $\overset{⁁}{R}$ is proportional to the noise covariance

(5) $$\overset{⁁}{\Psi }={\left\langle \left(\overset{⁁}{n}-{\left\langle \overset{⁁}{n}\right\rangle }_{\text{time}}\right){\left(\overset{⁁}{n}-{\left\langle \overset{⁁}{n}\right\rangle }_{\text{time}}\right)}^{†}\right\rangle }_{\text{time}}.$$

(Note that this is the conjugate of Eq. (A10) in 9.) Thus, in practice $\overset{⁁}{\Psi }$ is typically measured by digitizing >10^5^ noise samples $\overset{⁁}{n}$ (7, Eq. (5)) and substituting $\overset{⁁}{R}\to \overset{⁁}{\Psi }$ in Equation (4). The constant of proportionality is effectively absorbed into the constant C 7, 9, 24.

### Estimating B_1_^−^

Determining the receive fields $\overset{⁁}{b}(r_{k})$ is the challenging step in applying Equation (4). In this study, we test two approaches to *compute* $\overset{⁁}{b}(r_{k})$:

- In Roemer (BS B_1_^−^) combination, we use the known coil conductor geometry and the Biot‐Savart law to compute $\overset{⁁}{b}(r_{k})$ at zero frequency.
- In Roemer (BS B_1_^−^ phased) combination, we multiply by an additional single complex coefficient $\beta_{i}$ per element to account for preamp gains and phase shifts, cable losses and phase shifts, etc. The $\beta_{i}$ coefficients for each receive element were calibrated once using a uniform phantom (see Appendix 1).

### Recap of the WSVD Combination Algorithm

The WSVD combination algorithm 9 also uses Roemer's reception model (Eq. (2)) to process voxels independently:

(6) $$\bar{S}=\bar{\alpha }Q^{T}+\bar{\Xi }$$

where $\bar{S}$ is the $N_{i}\times N_{k}$ (no. coil elements × samples) matrix of single‐element spectra for a voxel, $\bar{\alpha }$ is the $N_{i}\times 1$ vector of coil sensitivities at that voxel, *Q* is the $N_{k}\times 1$ vector of spectral components of the sample magnetization in the voxel, and $\bar{\Xi }$ is white, multivariate normally distributed noise with covariance $\overset{⁁}{\Psi }$. The WSVD combination algorithm begins by applying a $N_{i}\times N_{i}$ noise‐whitening (decorrelation) matrix *W* to convert $\bar{S}$ into a matrix of whitened “channel” spectra (9, Eqs. (4), (5), (6)):

(7) $$S=W\bar{S}\text{where}W=D^{-1/2}X^{†}\text{and}XDX^{†}=\overset{⁁}{\Psi },$$

in which D contains the eigenvalues and X the eigenvectors of $\overset{⁁}{\Psi }$. Compared to our definition of W in 9, we omit an unnecessary 2 and take the conjugate transpose for compatibility with Equation (5).

The singular value decomposition (SVD) 27 gives:

(8) $$S=U\Sigma V^{†}$$

where U is an $N_{i}\times N_{i}$ unitary matrix whose columns are the left‐singular vectors, Σ is a diagonal matrix of singular values, and V is an $N_{k}\times N_{k}$ unitary matrix with columns that are the right‐singular vectors.

Finally, the first singular vectors ( $U_{•,1}$ and $V_{•,1}$) give the maximum likelihood coil sensitivities

(9) $$\bar{\alpha }=W^{-1}U_{•,1}/\phi$$

and combined spectrum

(10) $$Q=V_{•,1}^{*}\Sigma_{1,1}\times \phi ,$$

where ϕ is an arbitrary magnitude/phase term for each voxel. This term arises because none of the data‐driven methods in this study can determine the absolute magnitude or phase of the coil sensitivities; they only compute relative sensitivities because Equation (6) is undetermined.

### Link Between the WSVD and Roemer Combination Algorithms

We prove in Appendix 2 that the WSVD combination algorithm gives the same result as this two‐step process: 1) determine the maximum likelihood receive fields $\overset{⁁}{b}(r_{k})\propto {\bar{\alpha }}^{*}$; and 2) apply these in Roemer's formula (Eq. (4)) to obtain the maximum likelihood combined spectrum. (The WSVD combination algorithm actually performs these steps simultaneously in Eq. (8).)

In the Supporting Information, we recast the WSVD combination algorithm to obtain the optimal combination weights directly, i.e. without first using an SVD that also gives the optimal combined spectrum. It transpires that this is algebraically identical to Walsh's adaptive reconstruction algorithm for combining MR images 28.

### Asymptotic Optimality

In the high SNR limit, Equation (6) simplifies to $\bar{S}=\bar{\alpha }Q^{T}$, and the coil receive sensitivities computed during the WSVD combination algorithm become exact (apart from the per‐voxel overall phase/amplitude factor ϕ). In the high SNR limit, the WSVD combination algorithm is identically equivalent to the Roemer (Exact B_1_^−^) combination method. In MR imaging, this behavior has been termed *asymptotic optimality* 29, 30. We suggest that this is a minimum requirement for a “good” spectroscopy combination method.

The WSVD receive sensitivities become less accurate at lower SNR 31, 32, 33.

### Extended WSVD Combination Algorithms

We might be able to improve the WSVD combination algorithm at low SNR by using prior knowledge in the first step to improve the accuracy of the estimated receive fields $\overset{⁁}{b}(r_{k})\propto {\bar{\alpha }}^{*}$. The single‐element spectra can be treated aggressively in this first step, for example, by deleting parts of the spectra away from anticipated peaks, applying strong line broadening, or applying spatial blurring. The idea is to eliminate extraneous information in the data fed to the singular value decomposition so that it better estimates the receive fields.

In the second step, we combine the *original* single‐element spectra using these estimated receive fields in Equation A8. Hence, we obtain a final combined spectrum that is not truncated or broadened or spatially blurred. Yet, by Equation (4), this final spectrum has the maximum possible SNR if the receive fields that we determined in the first step are correct and the receive fields are as accurate as possible (i.e., they have maximum posterior probability) when taking as given both the data and our choice of prior knowledge in the first step 34.

For example, since we know the approximate linewidth of metabolite peaks in our spectrum, we can apodize the single‐element FIDs with a matched‐filter window function exp(−At) before Fourier transforming and applying Equations (6) through (9) to give $\overset{⁁}{b}(r_{k})\propto {\bar{\alpha }}^{*}$. Apodization suppresses high‐frequency noise, thereby improving the accuracy of the estimated coil sensitivities. We then use these improved coil sensitivities in Equation A8 to combine the original single‐element spectra. This WSVD+Apod combination algorithm improves coil combination without broadening the combined spectrum.

As a second approach, we know that the coil sensitivities are spatially smooth. So we may compute the coil sensitivities for a certain target voxel *k* by: 1) multiplying the single‐element spectra in *N_n_* neighbouring voxels *k*′ by weights $\chi_{k\prime }=\text{exp}(-|r_{k}-r_{k\prime }{|}^{2}/R)$, where R determines the extent of spatial blurring; 2) apodizing each weighted single‐element spectrum; 3) concatenating the weighted and apodized single‐element spectra from neighbouring voxels end‐to‐end to form a new $N_{i}\times N_{n}N_{k}$ matrix $\bar{S}$; 4) applying Equations (6) through (9) to give $\overset{⁁}{b}(r_{k})\propto {\bar{\alpha }}^{*}$; and 5) applying Equation A8 with the original single‐element spectra from voxel *k* only to give the combined spectrum. We call this the WSVD + Apod + Blur combination algorithm.

### Brown, RefPeak, and GLS Combination Algorithms

The Brown combination algorithm 13 is derived from Equation (4) by: using the conjugate of the first FID point 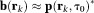 to approximate the receive sensitivities $\overset{⁁}{b}(r_{k})$ and neglecting noise, that is, assuming R≈I. This is analogous to Roemer's famous sum‐of‐squares method for MR imaging 4, 5. Siemens' (i.e. Siemens Healthcare, Erlangen, Germany) default spectroscopy combination algorithm is this variant of Brown combination:
(11)
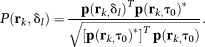
The Brown combination algorithm is asymptotically optimal if its assumption R≈I. holds.

Several coil combination methods have been proposed that involve fitting a reference peak in each single‐element spectrum and summing in proportion to the peak's amplitude, amplitude/noise standard deviation (SD), or amplitude/noise variance 9, 10, 14, 35, 36. Of these approaches, Hall's scaling by amplitude/noise variance 14 comes closest to asymptotic optimality. It is equivalent to neglecting off‐diagonal noise covariance matrix elements and using the conjugate of the fitted amplitudes to estimate $\overset{⁁}{b}(r_{k})$ in Equation (4). We implemented Hall's method 14 by: 1) fitting the single‐element spectra using the advanced method for accurate, robust, and efficient spectral fitting (AMARES); 2) multiplying each single‐element spectrum by the conjugate of the fitted complex amplitude /noise variance; and 3) summing. We call this the RefPeak combination algorithm.

The GLS combination algorithm 15 may be derived from Equation (4) by using the integral of the complex‐valued spectral signal over a reference peak to measure the coil sensitivities $\overset{⁁}{b}{\left(r_{k}\right)}^{*}$ and by using the spectral signal in a region where no metabolite peaks are detected to measure the noise covariance matrix 15. The GLS combination method is asymptotically optimal.

## METHODS

### Receive Array

**Figure 1:**
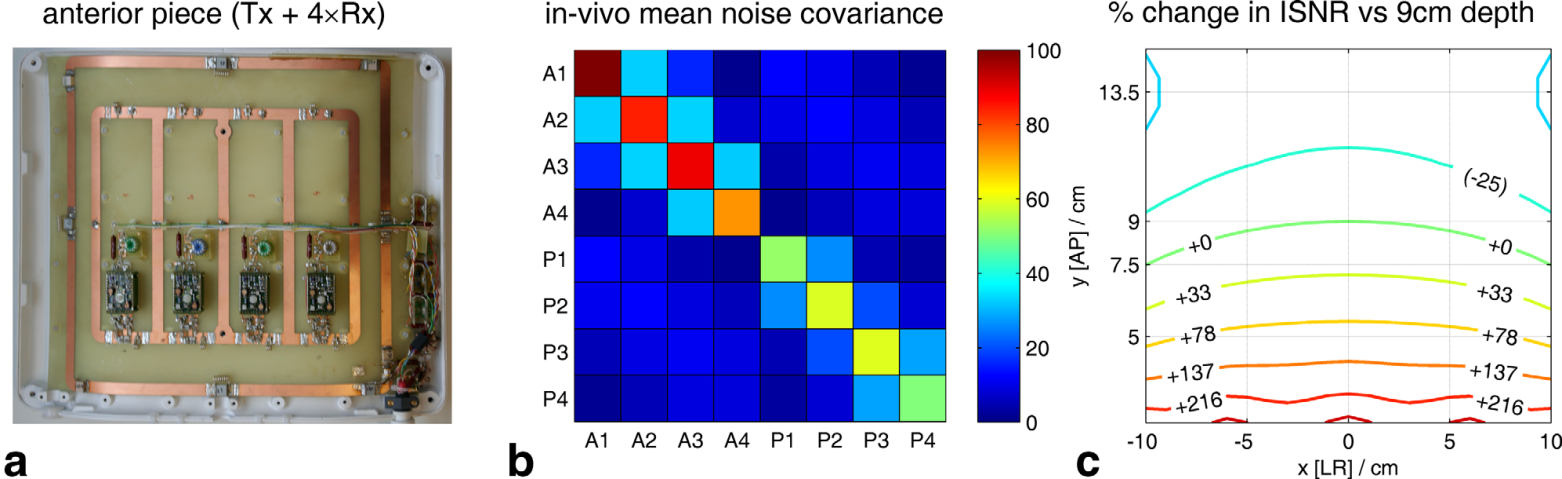
(a) Photograph of the anterior half of the eight‐element cardiac ^31^P 3T receive array coil employed in study. (b) Noise covariance matrix for this array. (Diagonal elements of the noise *covariance* matrix are the single‐coil noise variances. Note that this is not the same as the noise *correlation* matrix whose diagonal elements are all equal to 1 24.) (c) Contour plot of ISNR 24 in the midtransverse plane for the receive array with a 27‐cm separation between conducting elements. This plot is scaled relative to the interventricular septum (9‐cm depth). Horizontal lines mark the mean depth of the anterior mid‐short axis segments (7.5‐cm depth) and the inferior mid‐short axis segments (13.5 cm) across all subjects. A, anterior; ISNR, intrinsic signal‐to‐noise ratio; LR, left–right; P, posterior.

All data were acquired on a 3T Trio scanner (Siemens Healthcare, Erlangen, Germany) using the 8‐element cardiac ^31^P receive array shown in Figure 1a (Rapid Biomedical GmbH, Rimpar, Germany). The anterior piece of the array contains a 30 × 29‐cm^2^ transmit loop and four 6 × 20‐cm^2^ receive elements; the posterior piece contains four 6 × 20‐cm^2^ receive elements. The array's noise covariance matrix is shown in Figure 1b, and its intrinsic signal‐to‐noise ratio (ISNR) across the torso is shown in Figure 1c.

Four position markers (cod liver oil capsules) and a fiducial (consisting of a 1.8‐cm outer diameter plastic sphere (The Precision Plastic Balls Company Ltd, Ilkley, UK) filled with a solution of phenylphosphonic acid (PPA) and chromium (III) acetylacetonate (Cr(acac)_3_)) were fitted to the housing of each piece of the array. The anterior fiducial had ethanol solvent; the posterior had acetone solvent to give a distinct resonance 37.

During each scan, stacks of ^1^H images were acquired to locate the position markers and nonlocalized, inversion‐recovery–FID scans, with 2‐ms (anterior) and 10‐ms (posterior) inversion pulse duration, were acquired to monitor transmit efficiency. During each scan, a custom MATLAB (MathWorks, Natick, MA) tool was used to compute subject‐specific B_1_^±^ maps using the coil geometry, the Biot‐Savart law, and this calibration data. We acquired 204 800 noise samples from every receive channel in 5 s before each scan to determine the coil's noise covariance $\overset{⁁}{\Psi }$.

### Simulations

The receive array was simulated twice with CST Studio Suite 2013 (CST AG, Darmstadt, Germany), first adjacent to a 30 × 30 × 23‐cm^3^ uniform 73 mM NaCl phantom and then adjacent to the virtual human (Laura) voxel data (CST AG, Darmstadt, Germany). In both cases, the E‐ and H‐fields for each receive element were computed at 49.9 MHz (3T ^31^P) over a 3D volume with an open boundary using the finite‐differences time‐domain solver. The coil capacitors (4 × tune and 1 × match per loop) were adjusted for each run using Trust Region Framework optimization in CST Studio Suite 2013 to ensure S_11_ < −20 dB. The fields were imported into MATLAB (MathWorks) and used to determine B_1_^±^ according to Equations (1) and (3).

To evaluate the coil combination algorithms, we generated synthetic single‐element spectra for a slab by assuming a uniform metabolite concentration and using the CST (CST AG) human simulations for the true B_1_^−^ and adding noise. The noise covariance matrix was $\xi^{2}$ on the diagonal, $\xi^{2}/20$ between next‐neighbor coils, and zero elsewhere. We combined the synthetic spectra using each coil combination algorithm, fitted a Lorentzian peak to the combined spectra, and recorded the fitted amplitudes and phases. We repeated this procedure with 31 different noise levels ξ, repeating 20 times each. We simulated Biot‐Savart B_1_^−^ phase/gain coefficient calibration (see Appendix 1) by scaling the Biot‐Savart fields to match the CST (CST AG) simulation 10 cm below the center of the anterior piece of the array.

To test how the choice of combination algorithm affected the degree of signal contamination between neighbouring voxels, we performed a further simulation of a two‐compartment model. This comprised a slab of “skeletal muscle” (with phosphocreatine (PCr) to adenosine triphosphate (ATP) concentration ratio, PCr/ATP = 4.0) overlying “myocardium” (PCr/ATP = 2.0). Following An et al. 15, we also generated synthetic data for this model with an additional nonlocalized baseline artifact equal in each voxel to 2% of the PCr peak amplitude in the most sensitive coil. We repeated this procedure with 31 different noise levels, repeating 500 times each.

### Phantom

**Figure 2:**
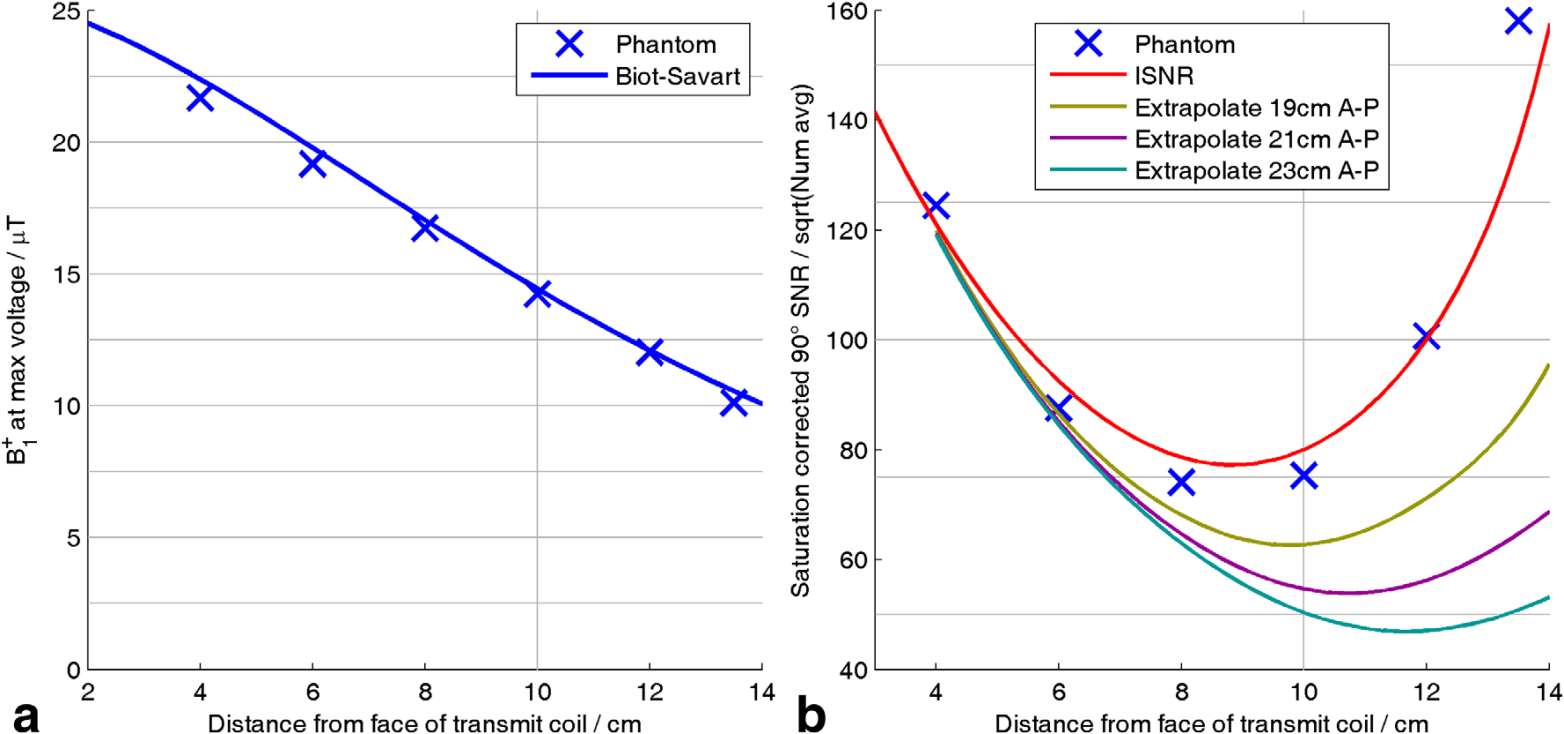
Validation of transmit and receive fields computed using the Biot‐Savart law against single‐depth experimental values from the phantom in Supporting Figure S1. (a) B_1_^+^ profile. Line shows B_1_^+^ computed using the Biot‐Savart law and nonlocalized inversion recovery data from the anterior fiducial. Each experimental value (*x*) is obtained by fitting a sinusoid to a series of fully relaxed, nonlocalized FIDs, with increasing flip angles acquired with the phosphate cube at that depth. (b) Experimental points show WSVD‐combined signal amplitude for fully‐relaxed 90° excitation at each depth. Red line shows corresponding ISNR values 24 computed at experimental coil separation (16.7 cm) and scaled vertically to fit experimental data. Other lines extrapolate experimental signals to model greater separations between anterior and posterior pieces of the array, which would be more representative of human subjects. AP, anterior–posterior; ISNR, intrinsic signal‐to‐noise ratio; WSVD, whitened singular value decomposition.

We validated the Biot‐Savart law B_1_^±^ calculations using a phantom 38 comprising a 2 × 2 × 2‐cm^3^ cube of KH_2_PO_4(aq)_ immersed in 14 L of saline (73 mM NaCl_(aq)_, i.e., conductivity matched to muscle 39, 40) (see Supporting Fig. S1). Figure 2a compares B_1_^+^ measured with the cube at six depths against the Biot‐Savart law B_1_^+^ map. Figure 2b compares the SNR for 90° excitation with the cube at six depths against the coil's ISNR.

To evaluate the coil combination algorithms and to calibrate the phase/gain coefficients for the Roemer (BS B_1_^−^ phased) combination method, we acquired 54 separate 3D chemical‐shift‐imaging (CSI) datasets from a 25 × 25 × 11‐cm^3^ uniform 38 mM K_2_HPO_4(aq)_ phantom (also conductivity matched to muscle 41, 42). We used a 30 × 30 × 20 ‐cm^3^ field of view, 16 × 16 × 16 matrix, acquisition weighting with 4 averages at k = 0, Hamming k‐space filtering, and repetition time (TR) = 3 s, giving a total duration of 10 h. The flip angle varied from 70° to 30° vertically through the center of the phantom. Summing all 54 repetitions yielded a gold standard dataset with sufficient SNR to fit the phosphate resonance from every receive element reliably for every voxel inside the phantom. The $\beta_{i}$ coefficients were obtained, as described in Appendix 1.

### In Vivo

Five volunteers (4 male + 1 female, 23–34 years, 65–82 kg, 1.70–1.93 m) were recruited in a manner approved by the local Research Ethics Committee. Cardiac ^31^P spectra were acquired specifically for this study using an established protocol 43, 44 with minimal modifications. Spectra were acquired with a 3D ultrashort echo‐time CSI pulse sequence 45, with acquisition weighting 46, 47 and electrocardiogram triggering, over a 35 × 35 × 3‐ cm^3^ field of view, with a CSI matrix of 22 × 22 × 10 voxels, and with 2 averages at k = 0. The total duration was 27 min at a heart rate of 70 bpm. Subjects were positioned head‐first supine (required for this coil). The subject‐specific B_1_^+^ maps were used to set the excitation voltage for a 30° flip angle in the center of the interventricular septum.

The standard Siemens reconstruction program for spectroscopy was modified to retain absolute phase information in the single‐element spectra, to store the prescan noise covariance matrix, and to implement the WSVD combination algorithm online. Single‐element spectra were also combined offline in MATLAB (MathWorks) .

### Analysis of Combined Spectra

Before examining the spectra, the left ventricle was segmented manually following the 17‐segment model 48. Spectra from voxels whose centers lay within a myocardial segment were automatically extracted for further analysis.

Spectra in each voxel were processed by: DC offset correction; estimation of frequency offset by cross‐correlation with a typical spectrum; and fitting with a custom MATLAB (MathWorks) implementation of AMARES 49, with prior knowledge specifying 11 Lorentzian peaks (α,β,γ‐ATP multiplet components, PCr, phosphodiester (PDE) and 2× 2,3‐diphosphoglycerate (2,3‐DPG)), with fixed amplitude ratios and scalar couplings for the multiplets. The fitted amplitudes were corrected for blood contamination by subtracting 30% of the average of the two 2,3‐DPG signals from each of the ATP amplitudes 50. The remaining PCr and ATP signals were corrected for saturation 51 using the Biot‐Savart B_1_^+^ map and literature T_1_ values 38. The final PCr/ATP ratio was:

(12) “PCr/ATP” = PCr/[(α‐ATP+β‐ATP+γ‐ATP)/3].

Cramér‐Rao lower bounds were computed 52, 53 from the AMARES parameter covariance matrix, assuming the flip angles and T_1_ values to be exact. Spectral SNR was defined as peak height/baseline SD after application of a matched filter 54.

## RESULTS

### Simulations

**Figure 3:**
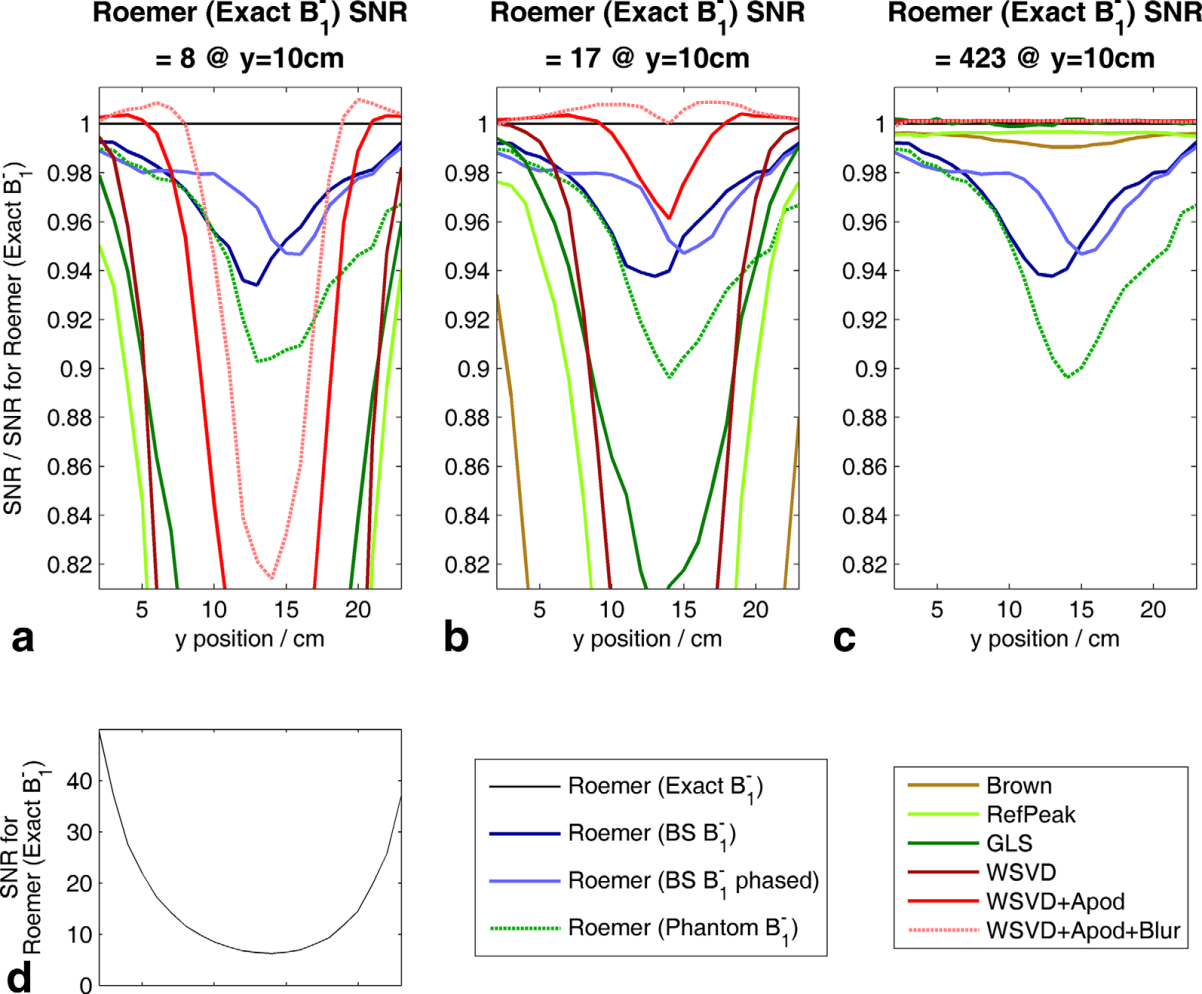
Performance of different coil combination algorithms applied to simulated spectra. The simulated receive array has 4× elements in the plane at y = 0 cm and 4× elements in the plane at y = 25 cm. Coordinate system is shown in Figure 5c; y is anterior‐posterior direction. (a–c) Mean SNR along a column from x = −10 to +10 cm at different depths. Values are plotted relative to SNR for Roemer (Exact B_1_^−^) combination. (d) Spatial variation of mean SNR for Roemer (Exact B_1_^−^) combination. Apod, apodized; BS, Biot‐Savart law; GLS, generalized least squares; SNR, signal‐to‐noise ratio; WSVD, whitened singular value decomposition.

Figure 3 summarizes the behavior of the combination algorithms applied to synthetic data by plotting 1D profiles of the combined signals at three noise levels. Note that the best possible SNR—from Roemer (Exact B_1_^−^) combination (Fig. 3d)—was greatest close to the receive elements, which lay at y = 0 cm and y = 25 cm. All methods performed better near the receive elements and broke down to varying extents deeper into the subject.

At high SNR (Fig. 3c), the three WSVD combination methods and the GLS combination method were practically perfect everywhere, which supports our assertion that they are asymptotically optimal. The Brown and RefPeak combination algorithms were almost as good, because $\overset{⁁}{\Psi }\approx I$ in the simulations, so they are almost asymptotically optimal here. Note, however, that the RefPeak combination algorithm was approximately one hundred times slower than the other methods. In contrast, the Roemer combination methods, using approximate B_1_^−^s, all showed a 5% to 10% drop in SNR near the middle of the subject. The Roemer combination methods performed worst here because the signal is split between low‐magnitude contributions in several of the elements. Hence, by the triangle inequality, errors in the relative phases of B_1_^−^ produce worse signal cancellation here than occurs close to the receive elements, where a few elements (or a single element) have dominating $\left|B_{1}^{-}\right|$. Finally, in the simulations, the Roemer (BS B_1_^−^ phased) combination $\beta_{i}$ coefficients were set; thus, B_1_^−^ was exact at (0, 10, 0) cm. Hence, the SNR increased appreciably compared to the SNR for the Roemer (BS B_1_^−^) combination method at ∼10‐cm depth, but at a cost of small decreases in SNR at depths < 7 cm and > 14 cm. The fact that the *relative* Biot‐Savart B_1_^−^s (i.e., the zero‐frequency values) cannot be made to match the true *relative* B_1_^−^s (i.e., the 49.9 MHz CST Studio (CST AG) values) at all points in space simultaneously shows that the true B_1_^−^ fields have a qualitatively different *shape* to Biot‐Savart B_1_^−^s rather than differing only by the single amplitude/phase scaling per element that we assumed for the Roemer (BS B_1_^−^ phased) combination method. We believe that these small deviations between the *shape* of the Biot‐Savart law B_1_^−^s and CST Studio (CST AG) B_1_^−^s are due to dielectric effects, as illustrated in Supporting Figure S2.

**Figure 4:**
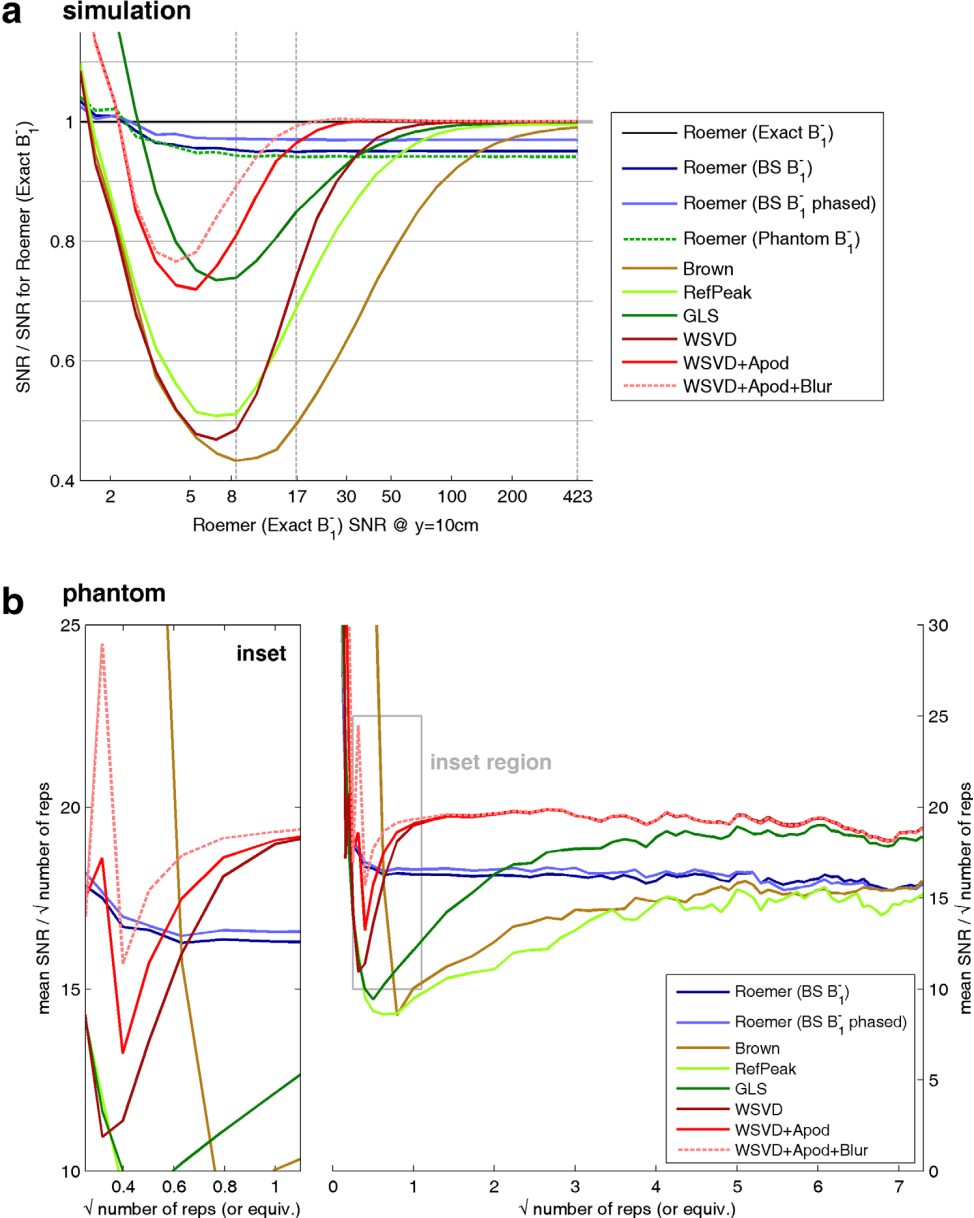
Summary of coil combination performance as a function of SNR. (a) Simulation. Each point on a line shows the mean over a 20 cm‐ (L–R) × 5‐cm (AP) region centered at 10‐cm depth (i.e., at the interventricular septum) of the SNR relative to the SNR for Roemer (Exact B_1_^−^) combination. Gray vertical lines mark the three SNR levels detailed in Figures 3a–c. (b) Phantom. Coil combination performance as a function of SNR in a uniform KH_2_PO_4(aq)_ phantom. Raw data comprise 54 three‐dimensional ultrashort echo‐time chemical‐shift imaging acquisitions. These were averaged in bunches to yield effective single‐element data with different SNRs, which were then combined. To better characterize the low‐SNR regime, further noise with the experimental covariance matrix was added before combination to give values with < 1 “repetitions”. Inset. Expanded view for low SNR. AP, anterior–posterior; Apod, apodized; BS, Biot‐Savart law; GLS, generalized least squares; LR, left–right; SNR, signal‐to‐noise ratio; WSVD, whitened singular value decomposition.

At low SNR (Figs. 3a–b), the performance of the Roemer combination methods was unchanged because these methods do not use the data to compute the B_1_^−^s. Meanwhile, the data‐driven methods began to break down as the estimated B_1_^−^s became insufficiently accurate. This is also evident in Figure 4a. Note that the rise at the left of Figure 4a reflects noise rectification when a Lorentzian peak is fitted to the combined spectra; it does not reflect the performance of the coil combination step.

Figure 4a shows that the data‐driven methods do not all fail at the same low SNR. We can rank them by the minimum SNR at which they outperform the Roemer (BS B_1_^−^) combination method. This is SNR ≈ 140 for the Brown combination method, 60 for the RefPeak combination method, 40 for the GLS combination method, 35 for the WSVD combination method, 15 for WSVD+Apod combination method, and 11 for the WSVD+Apod+Blur combination method.

**Figure 5:**
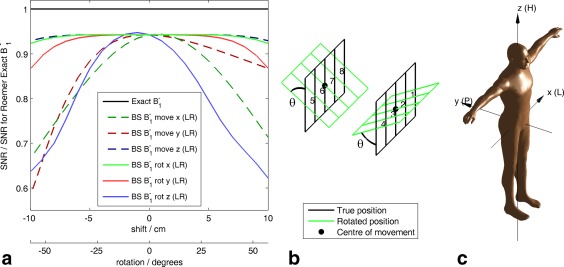
Sensitivity of Roemer (BS B_1_^−^) combination to misalignment of the coil relative to its expected location or orientation. For each point plotted, anterior and posterior pieces of the array were *either* translated by an equal and opposite amount parallel to the specified axis *or* rotated around the center of each piece by an equal and opposite angle around the specified axis. No noise was added during these calculations, that is, this figure corresponds to the high SNR limit at the right‐hand edge of Figure 4a. (a) Mean SNR relative to SNR for Roemer (Exact B_1_^−^) combination. (b) Diagram showing coil geometry for the “rot x” case. (c) Coordinate system employed 65. BS, Biot‐Savart law; H, head; L, left; P, posterior; R, right; SNR, signal‐to‐noise ratio.

A surface coil will typically be in a slightly different position every scan. To test if positioning errors will degrade the Roemer combination method in vivo, Figure 5 shows the effect of deliberately translating or rotating the receive array relative to its anticipated position on the Roemer (BS B_1_^−^) combination method. Rotations around the *x*‐ (LR) and *z*‐ (HF) axes and translations in the *y* (AP) direction are worst for this coil. Nevertheless, for this coil, errors due to this misalignment effect will be small, providing that the coil position is determined to within 1 cm.

**Figure 6:**
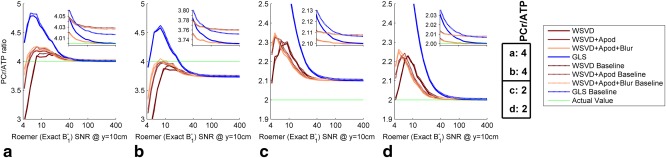
Simulation of coil combination performance in a two‐compartment phantom with a point spread function that gives 10% bleed from neighbouring voxels. Simulations were run 2000 times at each of 31 noise levels before averaging. Dotted lines show the effect of adding an extra 2% nonlocalized baseline “artifact” to the single‐element spectra before they are combined. Inset: Vertically magnified view of the same data for SNR from 40 to 400. Apod, apodized; GLS, generalized least squares; PCr/ATP, phosphocreatine to adenosine triphosphate concentration ratio; SNR, signal‐to‐noise ratio; WSVD, whitened singular value decomposition.

Figure 6 shows how the choice of coil combination method affects signal contamination from neighboring voxels in a two‐compartment model with finite voxel point‐spread‐function (PSF) and optionally also with a nonlocalized baseline artifact. In Figure 6a, we see that at high SNR, the GLS combination method is less susceptible to baseline artifact than the WSVD combination method, as previously reported by An et al. 15. However, in Figure 6b, the converse is true: the GLS combination method is more susceptible to baseline artifact. In Figures 6c and 6d, all combination methods show practically the same response to the baseline artifact. Further simulations (not shown) confirmed that the response of these methods to baseline artifacts depends on the particular situation. Overall, all of these methods have similar resistance to baseline artifacts. Comparing the three WSVD combination methods, the temporal and/or spatial apodization in the WSVD+Apod, and WSVD+Apod+Blur combination methods does not cause additional signal contamination in the combined spectra compared to the original WSVD combination method. The signal contamination is higher for the GLS combination method than for either the WSVD, WSVD+Apod, or WSVD+Apod+Blur combination methods in the low‐SNR regime in all cases.

### Phantom

Figure 4b plots the performance of each coil combination algorithm as a function of SNR in the uniform KH_2_PO_4(aq)_ phantom. At high SNR, the three WSVD combination methods and the GLS combination method outperformed the Roemer combination method; and the Brown and RefPeak combination methods were comparable to the Roemer combination method. This reflects the asymptotic optimality of the three WSVD combination methods and of the GLS combination method; the SNR penalty in the Brown and RefPeak combination methods now that $\overset{⁁}{\Psi }\neq I$; and the deviations of true B_1_^−^ from the Biot‐Savart calculations. At low SNR, the WSVD combination methods outperformed the Roemer (BS B_1_^−^) combination method when SNR > 14 (WSVD), SNR > 11 (WSVD+Apod), and SNR > 9 (WSVD+Apod+Blur). The Roemer combination methods perform comparably for all SNR. The Roemer (BS B_1_^−^ phased) combination method improves slightly over the Roemer (BS B_1_^−^) combination method. The Brown and RefPeak combination methods were worst at all SNR. This ranking of low‐SNR performances agrees with the simulations from Figure 4a.

**Figure 7:**
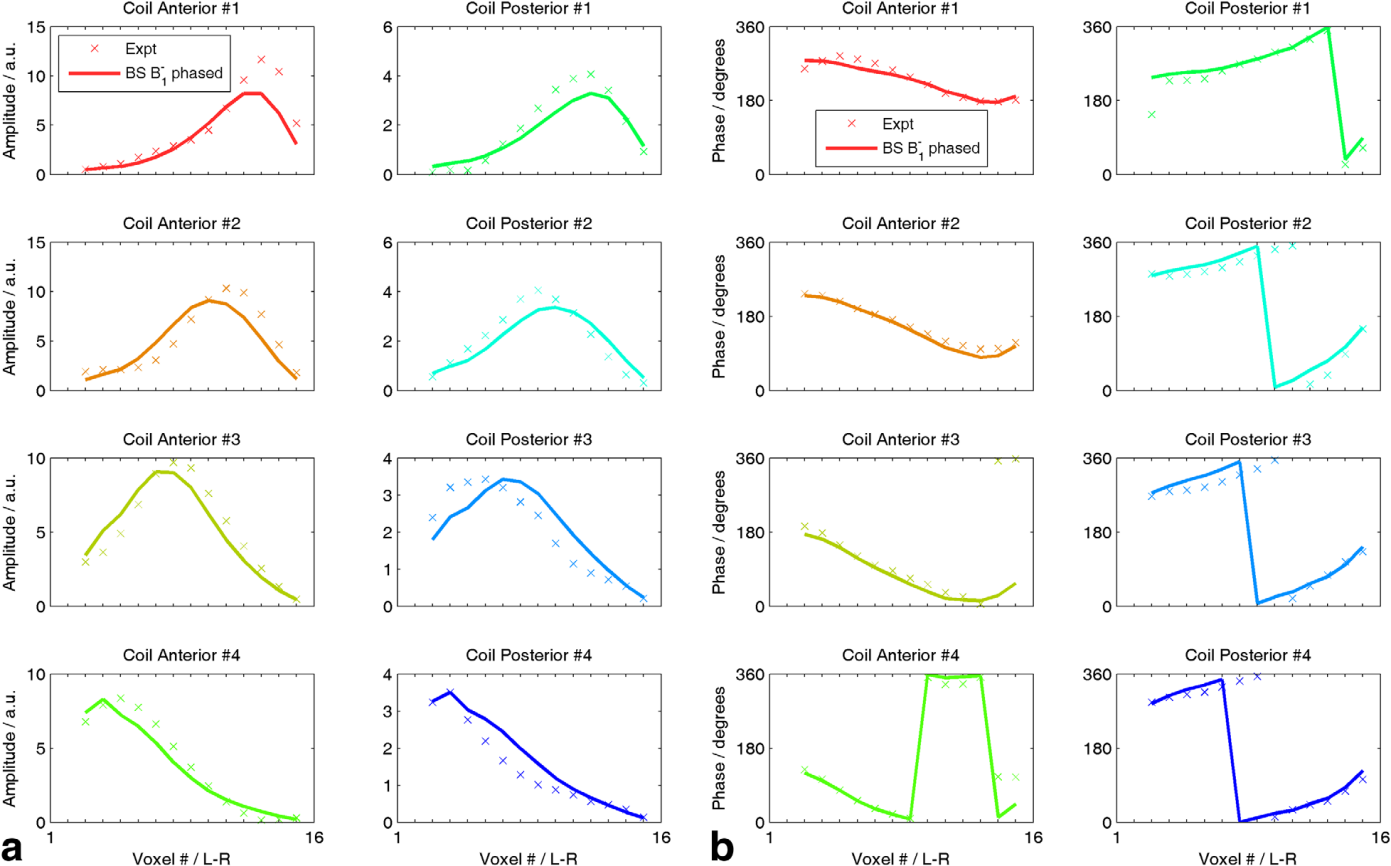
Illustration of phase/gain corrected B_1_^−^ fields from the procedure in Appendix 1. (a) Amplitudes and (b) phases for voxels running L‐R along center of uniform phantom. AMARES fits to the phosphate resonance are denoted *x* and the singular value decomposition best‐fit calculated B_1_^−^ is plotted with a line. BS, Biot‐Savart law; L‐R, left–right; AMARES, accurate, robust and efficient spectral fitting method.

The Biot‐Savart law phase/gain coefficients calibrated in the uniform phantom are listed in Table 1. The SVD quality factor Γ = 0.887 means that 89% of the power in the phantom data was explained by the Biot‐Savart field maps and the per‐element coefficient $\beta_{i}$. Figure 7 plots the experimental signals and predictions from Equation A(2). The mean $\left|\beta_{i}\right|$ was +18% (anterior) and −18% (posterior). The mean phase shift was 28° but followed no clear pattern.

**Table 1:** Numerical Results of the Roemer‐SVD Calibration Procedure on the Uniform Phantom

Coil Element	Amplitude Factor	Phase Factor/Degrees	SVD Quality Factor Γ
1	1.19	+14.79	0.887
2	1.12	−15.29	
3	1.20	−39.89	
4	1.19	−36.03	
5	0.81	+28.75	
6	0.75	+51.32	
7	0.82	+15.88	
8	0.91	−19.52	

SVD, singular value decomposition.

### In Vivo

As a further test of the Biot‐Savart fields, Supporting Table S1 compares $\left|B_{1}^{+}\right|$ measured at the posterior fiducial against $\left|B_{1}^{+}\right|$ calculated from the Biot‐Savart law. We observed differences of −4.3% to +18.8% for four subjects (the posterior fiducial SNR was inadequate in subject 5). These ∼10% errors are a little greater than in the phantom (Fig. 2).

**Figure 8:**
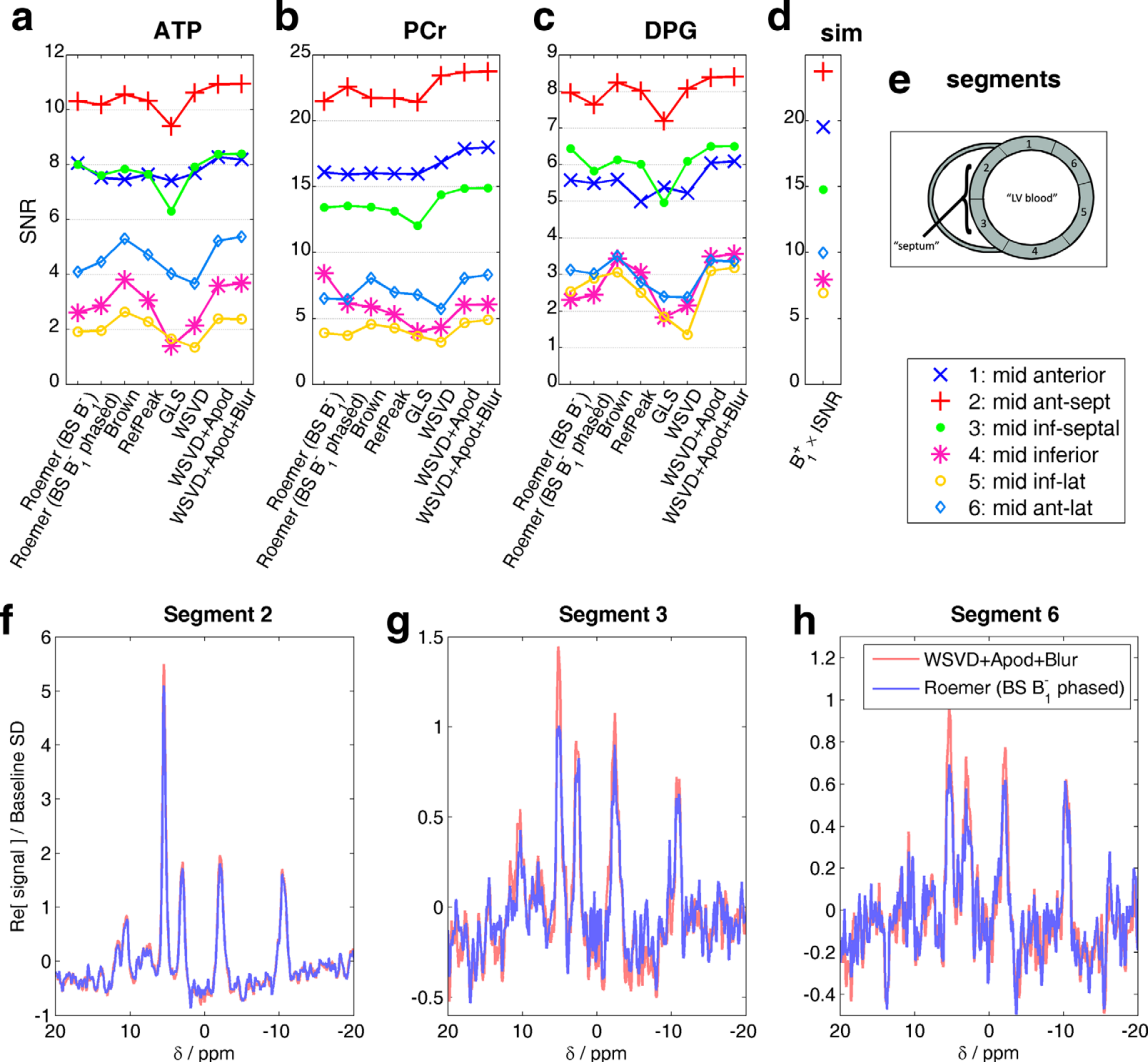
Comparison of mean metabolite SNRs after application of different coil combination algorithms for five normal volunteers. (a–c) Points show mean metabolite SNRs (average of α‐ATP, β‐ATP, γ‐ATP peak SNRs, PCr SNR, and average of 2,3‐DPG peak SNRs) in each mid‐short axis myocardial segment for each coil combination algorithm. Lines are only to guide eye. (d) B_1_^+^ × ISNR at the mean centroid of each segment. This value should be proportional to SNR in the limit of low flip angle and long repetition time. (e) Inset showing segment numbering 48. (f–h) Example spectra from a voxel in the centre of each segment combined with the best data‐driven and the best field map‐driven algorithms. Apod, apodized; BS, Biot‐Savart law; GLS, generalized least squares; PCr/ATP, phosphocreatine to adenosine triphosphate concentration ratio; DPG, 2,3‐diphosphoglycerate; SD, standard deviation; SNR, signal‐to‐noise ratio; WSVD, whitened singular value decomposition.

Figure 8 summarizes the metabolite SNRs from five normal volunteers. SNR depends primarily on the myocardial segment: signals are strongest in the mid‐anteroseptal, mid‐inferior septal, and mid‐anterior segments. Figure 8d plots $B_{1}^{+}\times \text{ISNR}$, which is proportional to SNR at low flip angle and long TR. $B_{1}^{+}\times \text{ISNR}$ agrees mostly with the SNR trends observed in Figures 8a to 8c, that is, the metabolite SNRs are dominated by the field profile of the RF coil.

Nevertheless, comparing the combination methods, we see that the WSVD+Apod and WSVD+Apod+Blur combination methods always yield higher SNR than the original WSVD combination method; the largest differences are in segments/metabolites with low SNR. This is consistent with simulations (Fig. 4a) and phantoms (Fig. 4b). In segments/metabolites with high SNR, such as PCr in the mid‐anteroseptal segment, the WSVD+Apod+Blur combination method gives ∼10% greater SNR than the Roemer (BS B_1_^−^) combination method, as expected. The Roemer (BS B_1_^−^ phased) combination method sometimes increases SNR compared to the Roemer (BS B_1_^−^) combination method and sometimes it lowers SNR; the calibration step appears to add little value in vivo.

Note that, although it appears that the Brown and RefPeak combination methods perform better than the WSVD combination method in segments/metabolites with the lowest SNR, this actually reflects the fact that the Brown and RefPeak combination methods have entered the lowest SNR limit where the apparent SNR is overestimated because of noise rectification during fitting. This effect was visible on the left of Figure 4. This means that, although the Brown and RefPeak combination methods appear superior in this low SNR limit, the opposite is actually true: the Brown and RefPeak combination methods have already broken down and become noise‐dominated, whereas the other methods have not.

**Figure 9:**
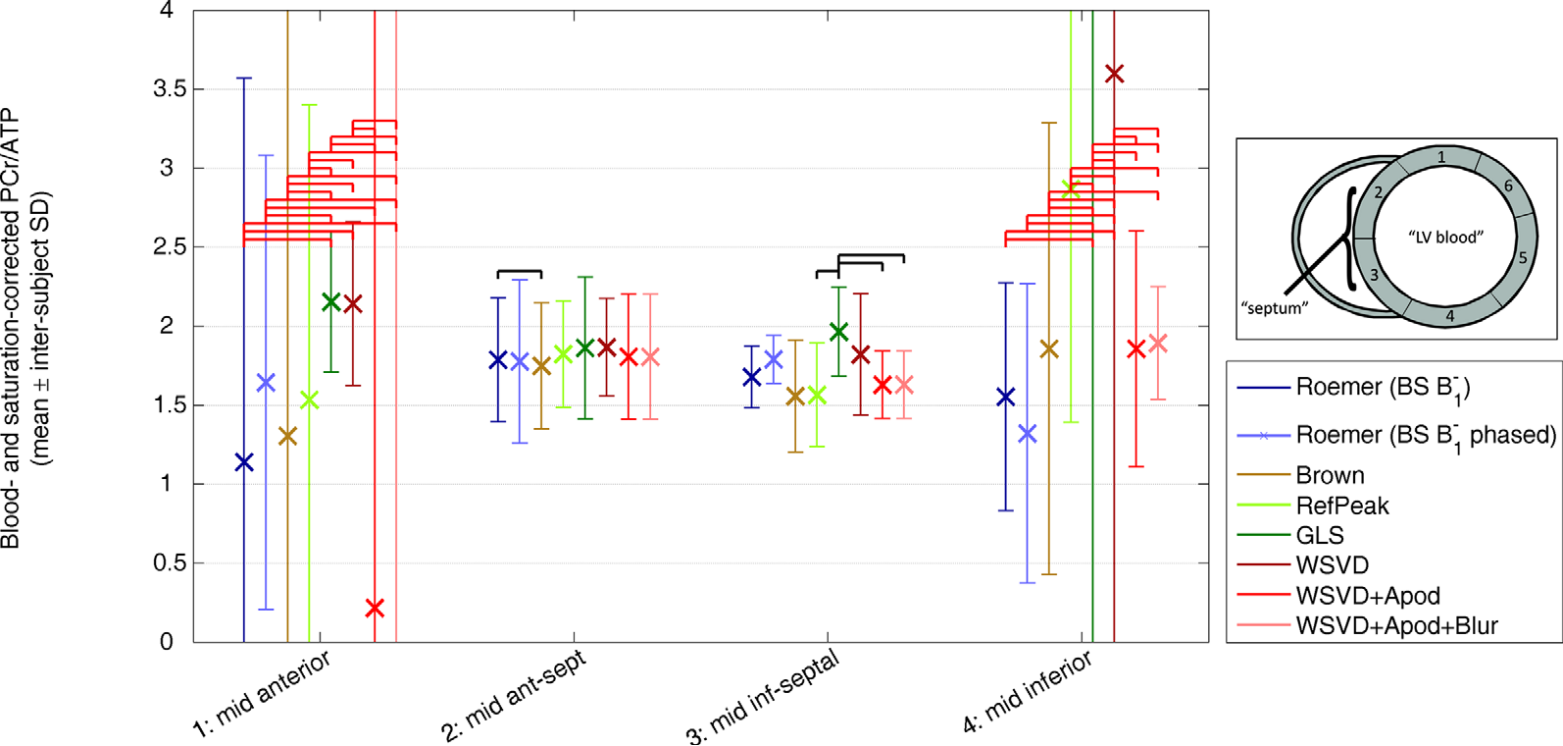
Comparison of blood‐ and saturation‐corrected PCr/ATP ratios for five normal volunteers. Points show intersubject mean metabolite ratio in each segment for each coil combination algorithm. Error bars show corresponding intersubject SD. Black horizontal bars indicate a statistically significant change in PCr/ATP ratio for different combination algorithms (i.e., *P* < 0.05 from a paired *t* test). Red horizontal bars indicate a statistically significant change in PCr/ATP variance (i.e., *P* < 0.05 from a paired two‐sample *F* test for equal variances). Apod, apodized; BS, Biot‐Savart law; GLS, generalized least squares; PCr/ATP, phosphocreatine to adenosine triphosphate concentration ratio; SD, standard deviation; WSVD, whitened singular value decomposition.

As well as increasing metabolite SNRs, it is important that a coil combination method be unbiased. To test for bias in vivo, Figure 9 plots the blood‐ and saturation‐corrected PCr/ATP concentration ratio in each segment for each combination algorithm. All the combination algorithms produced consistent and plausible metabolite ratios in the septal segments. Statistically, significant differences in the mean PCr/ATP ratio were observed only once in the mid‐anteroseptal segment and three times in the mid‐inferior septal segment. In the mid‐anterior and mid‐inferior segments, the intersubject variances showed statistically significant differences in almost all cases, but there were no statistically significant differences in the mid‐anteroseptal and mid‐inferior septal segments.

## DISCUSSION

### Accuracy of Field Maps

The Roemer combination methods depend critically on the quality of their $B_{1}^{-}$ maps. Experimentally, we tested two approaches to obtain these: 1) using the Biot‐Savart law (BS B_1_^−^) and 2) using the Biot‐Savart law *and* multiplying by a phantom‐calibrated phase/gain coefficient per element (BS B_1_^−^ phased). Both approaches were worse than the data‐driven WSVD, WSVD+Apod, and WSVD+Apod+Blur combination methods, except at SNRs so low that the combined data was unsuitable for further analysis anyway. This implies that Biot‐Savart B_1_^−^s were not accurate enough to compete with B_1_^−^s inferred from the data during the WSVD, WSVD+Apod, and WSVD+Apod+Blur combination methods.

Another popular approach is to determine sensitivity maps for the coil directly from a uniform phantom. This requires a phantom that loads the coil so as to reproduce accurately the in vivo $B_{1}^{-}$. Our simulations (Fig. 4a) suggest that, to perform better than the WSVD combination method, this approach requires a more sophisticated choice of phantom than our uniform cuboid.

Finally, for ^1^H‐MRSI, good results are obtained by using weak water suppression and using the residual water peak as a high SNR internal reference for coil combination 9, 13, 55. Good results also arise by using prescan ^1^H images to infer accurate B_1_^−^ maps, which can be used to combine subsequent MRSI single‐element spectra with the multichannel spectroscopic data combined by matching image calibration data algorithm 56.

### Are Biot‐Savart Field Maps the Right Shape?

We corrected the Biot‐Savart field maps using a complex scaling $\beta_{i}$ per‐element in the Roemer (BS B_1_^−^ phased) combination method. However, this hardly improved the combined SNR compared to Roemer (BS B_1_^−^) combination. This could be because our phantom does not load the coil like a human subject, so our $\beta_{i}$ values are not quite right.

To test the sensitivity of $\beta_{i}$ to loading, we inspected the virtual human (Laura), CST (CST AG) B_1_^−^s at the centroid of each receive element. The amplitude of B_1_^−^ varied by −40% to +55% (vs. mean) and the phase by −9° to +9°. This suggests that changes in coil loading between subjects will be significant.

We therefore adapted the $\beta_{i}$ calibration to run directly on human data using alternating least squares (see Supporting Information). When applied to voxels covering the left ventricle (not shown), this actually gave slightly lower SNR than the phantom‐calibrated Roemer (BS B_1_^−^ phased) combination method.

This shows that the Biot‐Savart calculated fields differ somewhat in shape from the actual fields in vivo rather than simply by a per‐element complex scaling. This could be due to the presence of capacitors in the coil or dielectric effects in the subject.

### Ultrahigh Field

This work was performed at 3 T (i.e., 49.9 MHz), with sample dimensions appropriate for human cardiac studies. As field strength increases, dielectric effects 57, 58, 59 increasingly distort B_1_^−^ and B_1_^−^ fields, making them different to values at zero frequency (i.e., from the Biot‐Savart law). These distortions are approximately proportional to distance, conductivity, magnetic permeability, and the Larmor frequency 60, 61, 62, 63. Further, CST (CST AG) simulations in Supporting Figure S2 showed that the B_1_^+^/B_1_^−^ ratio from a 20 × 6‐cm^2^ loop varies by ∼20% across the heart and lungs at 49.9 MHz. For ^31^P‐MRS at 3 T, this has a minimal effect on the SNR of Roemer (BS B_1_^−^)‐combined spectra; but at higher fields, dielectric effects will make data‐driven coil combination methods even more preferable to methods based on B_1_^−^ maps.

### Breakdown of the Point‐Source Signal Model

The methods here all assume that the single‐element spectra for each voxel arise from a volume of space with a unique $B_{1}^{-}$ per element. The magnetization is therefore scaled in Equation (6) by a single complex coefficient per element to obtain the single‐element spectra. This model implies that *any* weighted sum of the single‐element spectra gives the *same* peaks, line shapes, relative amplitudes, and relative phases in the combined spectrum; the weighting coefficients only determine the level of noise and the overall complex scaling of the spectrum. For example, in ^31^P‐MRS, the commonly reported PCr/ATP ratio ought to be unchanged for any choice of weighting coefficients, although one choice will minimize the uncertainty of the PCr/ATP ratio. This can be observed in Figure 9. In segments 2 and 3, the PCr/ATP ratio is almost identical for all the combination methods, but the SDs vary.

Of course, this is only an approximation: all localization methods have a finite PSF, so each voxel contains contributions from a volume that in general does not have constant B_1_^−^s. Other artifacts, such as macromolecular baseline corruption, lipid contamination, or physiological motion, may allow signals arising outside the prescribed voxel to contaminate the single‐element spectra. Therefore, when processing experimental data, it may be better to employ a combination algorithm that suppresses such artifacts, even if it sacrifices some SNR in the combined spectra. Figure 6 suggests that the WSVD, WSVD+Apod, and WSVD+Apod+Blur combination methods are comparable to the GLS combination method in suppressing these artifacts, and that that the temporal and/or spatial apodization in the WSVD+Apod and WSVD+Apod+Blur combination methods does not amplify such artifacts.

### Practical Experience of the WSVD Combination Method

We have used the WSVD combination method routinely during the last 4 years. The WSVD combination method adds only ∼5 s per subject for acquiring a noise scan and only ∼2 s to online reconstruction of large CSI datasets (32 × 32 × 16 matrix; 2,048 samples; 8 channels). Application in vivo has proved to be trivial.

The only problem we have observed occurs if a low resolution CSI acquisition also excites extremely strong signals, for example, coil fiducials. This strong signal can blur into the target voxels at a level comparable to the true metabolite signals, which violates the point source model in Equation (6). This can cause the WSVD combination method to compute suboptimal weights. To avoid this, either 1) reduce bandwidth so the contaminating signals are not recorded; or 2) zero the single‐element spectra for the range of chemical shifts around the contaminating signal before applying the WSVD combination method.

In the spirit of reproducible research, we provide a MATLAB (MathWorks) implementation of these WSVD combination methods in the file “wsvd.m” in the Supporting Information and will share source code for a Siemens online image calculation environment (Siemens Healthcare) implementation subject to the normal formalities.

## CONCLUSION

For receive array spectra acquired from torso‐sized objects at frequencies ≥ 49.9 MHz, the data‐driven methods considered here all outperform, for any reasonable SNR, combination using coil sensitivities computed by the Biot‐Savart law in Roemer's formula. This is because the data‐driven methods effectively infer, from the data, coil sensitivities that rapidly become more accurate than computed B_1_^−^s as the single‐element SNRs increase.

We also introduced two novel methods: the WSVD+ Apod and WSVD+Apod+Blur combination methods. These out‐performed the Brown, RefPeak, and GLS combination methods at moderate‐to‐low SNR.

We tested these methods in vivo using human cardiac ^31^P‐MR spectra at 3 T, showing improvements in SNR compared to other combination methods and a few statistically significant changes in the PCr/ATP ratio due to the choice of coil combination method.

The theory presented here is equally applicable to other nuclei, in other organs, and at other field strengths. These new methods are therefore another step toward routine use of receive arrays for spectroscopy, particularly for X‐nuclei.

## Supporting information


**Figure S1.** Flip angle calibration. Phantom used for B_1_^+^ and B_1_^–^ measurements. Phantom is a Perspex box with outer dimensions 46 × 24 × 16.7‐cm^3^. It contains 14 L of 73 mM saline for loading, and a height‐adjustable 2 × 2 × 2‐cm^3^ cube of KH_2_PO_4(aq)_ gives the only ^31^P signal. The phantom rests on the anterior piece of the receive array and the posterior piece of the receive array goes on top.
**Figure S2.** Computed dielectric effects at 49 MHz. Ratio of |B_1_^+^| / |B_1_^−^| computed using CST Studio 2014 (CST AG, Darmstadt, Germany) and the Laura virtual human voxel model for one of eight receive elements at 2 MHz and at 49.9 MHz. At zero frequency, the Biot‐Savart law shows that $B_{1}^{\text{BS}}=B_{1}^{+}=B_{1}^{-}{\;}^{*}$, so this magnitude ratio does not vary with position at zero frequency. At higher frequency, dielectric effects can make this ratio vary with position. In this figure, there is ∼2% variation across the Laura model's torso at 2 MHz and ∼20% variation at 49.9 MHz. Dielectric effects at 49.9 MHz are small, but sufficient to cause modest effects on the SNR of Roemer‐combined spectra and comparable to those we observed in simulations, phantoms, and in vivo. SNR, signal‐to‐noise ratio.
**Table S1.** Biot‐Savart B_1_^−^ Fields In Vivo.

Supporting Information

## APPENDIX 1 Per‐Element Gain/Phase Calibration

We assume that the fitted complex amplitudes $s_{i}(r_{k})$ in the phantom for coil i in voxel k satisfy

(A1) $$s_{i}(r_{k})=\beta_{i}^{*}B_{1,i}^{-}{\left(r_{k}\right)}^{*}M_{xy}(r_{k}),$$

using the notation defined in the Theory section. Discretizing over voxels with strong signal gives

(A2) $$s_{ik}=\beta_{i}^{*}B_{1,ik}^{-*}m_{k}.$$

Using the Biot‐Savart law to approximate the sensitivities gives

(A3) $$s_{ik}^{*}/{\tilde{B}}_{1,\text{BS},ik}^{-}=X_{ik}\approx B_{i}m_{k}^{*}.$$

That is, we seek coefficients $\beta_{i}$ to optimize this rank‐1 approximation of **X**. If the noise in all elements of **X** is negligible, then using the singular value decomposition $X=U\Sigma V^{†}$ 27, 32, the optimal solution is

(A4) $$\beta_{i}=U_{•,1}\;\;\;\;\;\text{and}\;\;\;m_{k}\;\;\;=\;\;\Sigma_{1,1}V_{•,1}.$$

Thus, in Equation (4), we substitute $b\to \tilde{b}$, where these “BS B_1_^−^ phased” fields are

(A5) $${\tilde{b}}_{i}(r_{k})=\beta_{i}B_{1,\text{BS},i}^{-}(r_{k}).$$

Accounting for nonnegligible noise, as occurs in vivo, requires a more sophisticated approach, such as the alternating least squares method in the Supporting Information.

## APPENDIX 2 The WSVD and Roemer Combination Methods

To prove that the whitened singular value decomposition (WSVD) and Roemer combination methods are intimately related, we rearrange Equation (8) to give

(A6) $$V=S^{†}U\Sigma^{-1}\Rightarrow V_{•,1}=S^{†}U_{•,1}/\Sigma_{1,1}.$$

Combining with Equation (10), we write:

(A7) $$Q=S^{T}U_{•,1}^{*}\phi ={\bar{S}}^{T}W^{T}U_{•,1}^{*}\phi .$$

In other words, we have shown that the maximum likelihood combined spectrum takes the form of a linear combination of the single‐channel spectra.

To see whether this is the *same* linear combination that is prescribed by the Roemer combination method, we substitute Equation (9) to find

(A8) $$\begin{array}{l} Q={\bar{S}}^{T}W^{T}(W\bar{\alpha }\phi ){\;}^{*}\phi \\ \;\;\;\;\;={\bar{S}}^{T}(\Psi^{-1}){\;}^{*}{\bar{\alpha }}^{*}|\phi {|}^{2} \end{array}$$

(using the identities: $W^{-1}=XD^{+1/2}$, $WW^{†}=D^{-1}$, $W^{†}W={\overset{⁁}{\Psi }}^{-1}$, $W\overset{⁁}{\Psi }W^{†}=I$ and $\alpha =W\bar{\alpha }$).

If we choose to normalize $1=|\alpha |=\sqrt{\alpha^{†}\alpha }=\sqrt{{\bar{\alpha }}^{†}W^{†}W\bar{\alpha }}=\sqrt{{\bar{\alpha }}^{†}\Psi^{-1}\bar{\alpha }}$, we can further write

(A9) $$Q=|\phi {|}^{2}\frac{{\bar{S}}^{T}{\left(\Psi^{-1}\right)}^{*}{\bar{\alpha }}^{*}}{\sqrt{{\bar{\alpha }}^{T}{\left(\Psi^{-1}\right)}^{*}{\bar{\alpha }}^{*}}}.$$

Comparing with Equation (4), we observe that:

1. $\overset{⁁}{p}(r_{k},\delta_{l})=\bar{S}$ because they are both defined as the matrix containing the single‐element spectra (in Eqs. (4) and (6), respectively);
2. ${\overset{⁁}{R}}^{-1}=k_{b}\Psi^{-1}$, where $k_{b}$ is a collection of constants, which Brown et al. proved using the fluctuation‐dissipation theorem and verified experimentally 64; and
3. $\overset{⁁}{b}(r_{k})=k_{c}{\bar{\alpha }}^{*}$, when the data has high SNR, where $k_{c}$ is another collection of constants. This follows by comparing the Hoult definition of MR signal in Equation (2) to the WSVD signal model in Equation (6), and using Wedin's theorem to confirm the accuracy of the singular vectors at high SNR 31, 32, 33.

Substituting (a–c) into Equation (4) and using Equation A9, we find that

(A10) $$\begin{array}{l} \overset{⁁}{P}(r_{k,}\delta_{l})=C\frac{{\bar{S}}^{T}{\left(k_{b}\Psi^{-1}\right)}^{*}k_{c}{\bar{\alpha }}^{*}}{\sqrt{k_{c}^{*}{\bar{\alpha }}^{T}{\left(k_{b}\Psi^{-1}\right)}^{*}k_{c}{\bar{\alpha }}^{*}}}\\ \;\;\;\;\;\;\;\;\;\;\;\;\;\;\;\;\;\;=C\sqrt{\frac{k_{b}^{*}k_{c}}{k_{c}^{*}}}\frac{{\bar{S}}^{T}{\left(\Psi^{-1}\right)}^{*}{\bar{\alpha }}^{*}}{\sqrt{k_{c}^{*}{\bar{\alpha }}^{T}{\left(k_{b}\Psi^{-1}\right)}^{*}k_{c}{\bar{\alpha }}^{*}}}\\ \;\;\;\;\;\;\;\;\;\;\;\;\;\;\;\;\;\;\;=\frac{C}{|\phi {|}^{2}}\sqrt{\frac{k_{b}^{*}k_{c}}{k_{c}^{*}}}Q.\; \end{array}.$$

So, at high SNR, spectra combined with the WSVD and Roemer combination methods are equal, except that they may have different (arbitrary) overall magnitude and phase. At lower SNR, Wedin's theorem 31, 32, 33 shows that the WSVD ${\bar{\alpha }}^{*}$ begin to deviate from the true $\overset{⁁}{b}(r_{k})$. Nevertheless, the WSVD‐combined spectrum is still the same as what was obtained using the (inaccurate) WSVD‐estimated $\overset{⁁}{b}(r_{k})$ in the Roemer formula.
