# Supplementary material for: Coil combination for receive array spectroscopy: Are data‐driven methods superior to methods using computed field maps?
Source: Magn Reson Med. 2015 Mar 28;75(2):473–87. doi: 10.1002/mrm.25618 (PMC4744755; doi:10.1002/mrm.25618)
Supplement: Supplementary file 1 — Figure S1. Flip angle calibration. Phantom used for B1 + and B1 – measurements. Phantom is a Perspex box with outer dimensions 46 × 24 × 16.7‐cm3. It contains 14 L of 73 mM saline for loading, and a height‐adjustable 2 × 2 × 2‐cm3 cube of KH2PO4(aq) gives the only 31P signal. The phantom rests on the anterior piece of the receive array and the posterior piece of the receive array goes on top. Figure S2. Computed dielectric effects at 49 MHz. Ratio of |B1 +| / |B1 −| computed using CST Studio 2014 (CST AG, Darmstadt, Germany) and the Laura virtual human voxel model for one of eight receive elements at 2 MHz and at 49.9 MHz. At zero frequency, the Biot‐Savart law shows that B1BS=B1+=B1− *, so this magnitude ratio does not vary with position at zero frequency. At higher frequency, dielectric effects can make this ratio vary with position. In this figure, there is ∼2% variation across the Laura model's torso at 2 MHz and ∼20% variation at 49.9 MHz. Dielectric effects at 49.9 MHz are small, but sufficient to cause modest effects on the SNR of Roemer‐combined spectra and comparable to those we observed in simulations, phantoms, and in vivo. SNR, signal‐to‐noise ratio. Table S1. Biot‐Savart B1 − Fields In Vivo. [file MRM-75-473-s001.docx]

# Supporting Information

# for

# “Coil combination for receive array spectroscopy: are data-driven methods superior to methods using computed field maps?”

Christopher T. Rodgers and Matthew D. Robson

## Flip angle calibration

**
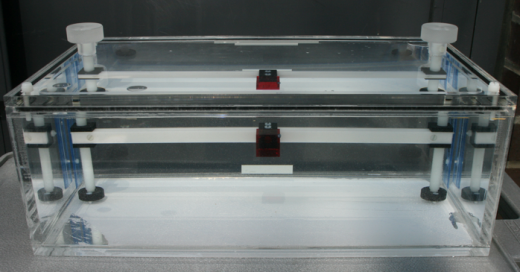
**

**Supporting Figure S1**: Phantom used for B_1_^+^ and B_1_^–^ measurements. The phantom is a Perspex box with outer dimensions 46x24x16.7cm^3^; it contains 14L of 73mM saline for loading; and a height-adjustable 2x2x2cm^3^ cube of KH_2_PO_4(aq)_ gives the only ^31^P signal. The phantom rests on the anterior piece of the receive array and the posterior piece of the receive array goes on top.

## Computed dielectric effects at 49MHz

| 2MHz | 49.9MHz |
| --- | --- |
| 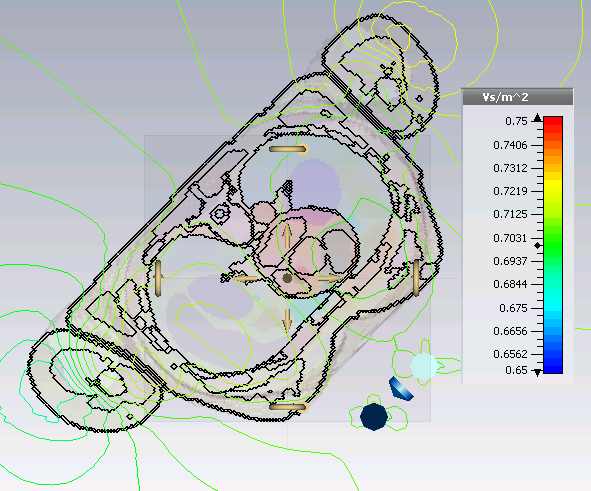 | 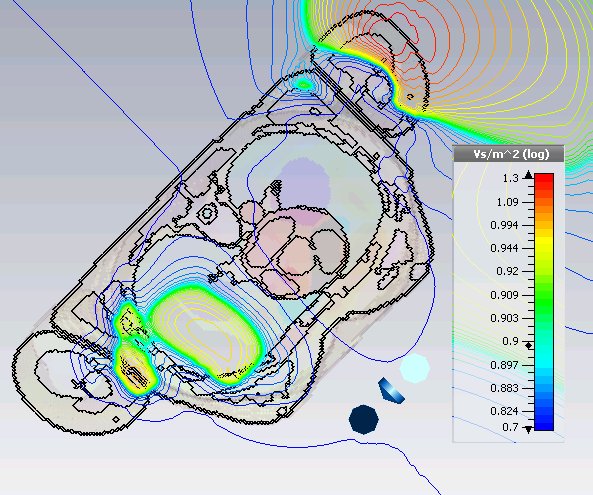 |

**Supporting Figure S2**: Ratio of |B_1_^+^ | / |B_1_^-^ | computed using CST Studio 2014 and the Laura virtual human voxel model for one of the eight receive elements at 2MHz and at 49.9MHz. At zero frequency, the Biot-Savart Law shows that $B_{1}^{\text{BS}}=B_{1}^{+}=B_{1}^{-*}$ so this magnitude ratio does not vary with position at zero frequency. At higher frequency, dielectric effects can make this ratio vary with position. In the plots above, there is ~2% variation across “Laura”’s torso at 2MHz and a ~20% variation at 49.9MHz. Dielectric effects at 49.9MHz are small, but sufficient to cause modest effects on the SNR of Roemer-combined spectra SNR, comparable to those we observed in simulations, phantoms and in-vivo.

## Biot-Savart B_1_^-^ fields in vivo

| **Measured IR FID anterior reference / V** | **Measured IR FID posterior reference / V** | **Biot-Savart predicted posterior reference / V** | **% error in predicted posterior B_1_^+^** |
| --- | --- | --- | --- |
| 460 | 3647 | 3070 | +18.8 |
| 397 | 3627 | 3346 | +8.4 |
| 403 | 3611 | 3674 | -1.7 |
| 403 | 3569 | 3730 | -4.3 |

**Supporting Table S1**: Comparison of reference voltages measured at the fiducials in vivo to a Biot-Savart Law extrapolation using the anterior fiducial to predict the reference voltage at the posterior fiducial. The "reference voltage" is defined as the voltage required for a 1ms hard pulse to give a 180° nutation. In other words, γB_1_^+^ = 500 Hz at the reference voltage.

## Appendix SI1: Further Theory

### Relationship between “noise resistance” R and “noise covariance” Ψ

When a receive array is used, the voltage noise in each element is almost exactly white around the Larmor frequency, with a multi-variate normal distribution characterised by the noise covariance matrix $\Psi$. The theory of RF coils interprets this noise as Johnson noise from a hypothetical resistive-capacitative-inductive (RLC) network. Brown *et al.* (1) summarised the current understanding of noise in magnetic resonance.

In practical terms, when comparing results from different sources, care must be taken to check which convention is used for the noise covariance matrix i.e. (2, Eq. 5) or (3, Eq. 22):

$\hat{\mathbf{\Psi}}=\left\langle\left(\mathbf{n}-\left\langle\mathbf{n}\right\rangle_{\text{time}}\right)\left(\mathbf{n}-\left\langle\mathbf{n}\right\rangle_{\text{time}}\right)^{\dagger}\right\rangle_{\text{time}} \text{or} \hat{\mathbf{\Psi}}=\left\langle\left(\mathbf{n}-\left\langle\mathbf{n}\right\rangle_{\text{time}}\right)^{*}\left(\mathbf{n}-\left\langle\mathbf{n}\right\rangle_{\text{time}}\right)^{T}\right\rangle_{\text{time}}.$ [SI.1]

When referring to coil literature, the noise resistance matrix $\hat{\mathbf{R}}$ is often used instead of the noise covariance matrix $\hat{\mathbf{\Psi}}$. According to Brown (1), $\hat{\mathbf{\Psi}} \propto\hat{\mathbf{R}}$, so the two quantities are often used interchangeably. However, again, care must be taken regarding the phase of the off-diagonal elements of $\hat{\mathbf{R}}$ to ensure these match the convention chosen for $\hat{\mathbf{\Psi}}$.

In Roemer’s seminal paper on receive arrays, he defines (his Eq [9])

$R_{ik}=\sigma\int\mathbf{E}_{i}\left(x,y,z\right)\bullet\mathbf{E}_{i}\left(x,y,z\right)dV.$ [SI.2]

Since $\mathbf{E}$ is a phasor (complex) variable, the imaginary part of this integral is in general non-zero. However, Roemer chose to ignore the imaginary part of this integral and therefore later assumed that R is symmetric. However, strictly, $\hat{\mathbf{R}}$ and $\hat{\mathbf{\Psi}}$ are complex Hermitian matrices by symmetry of the dot product (4 §10.4).

## Validation of Eq. [4] as written

The Roemer combination method (Eq. [4]) may also be thought of comprising two steps: (i) noise whitening; and (ii) summation of the whitened channel spectra weighted by their receive sensitivities to give the maximum SNR combined spectrum. To see this, we substitute $\hat{\mathbf{R}}\to\hat{\mathbf{\Psi}}$ in Eq. [4] and apply the identify $\hat{\mathbf{\Psi}}^{-1}=W^{\dagger}W$ to give:

$\hat{P}\left(\mathbf{r}_{k},\delta_{l}\right)=C\frac{\left[W\hat{\mathbf{p}}\left(\mathbf{r}_{k},\delta_{l}\right)\right]^{T}\left[W^{*}\hat{\mathbf{b}}\left(\mathbf{r}_{k}\right)\right]}{\sqrt{\left[W^{*}\hat{\mathbf{b}}\left(\mathbf{r}_{k}\right)\right]^{\dagger}\left[W^{*}\hat{\mathbf{b}}\left(\mathbf{r}_{k}\right)\right]}}.$ [SI.3]

The matrices $W$ act inside each square bracket to change basis from receive elements with correlated noise to "channels" with de-correlated noise of unit variance. This can be seen by noting that the top-left bracket matches Eq. [7] and by noting that the MR signal $s\propto\left(B_{1}^{-}\right)^{*}\hat{M}_{xy}\left(\mathbf{r}_{k}\right)$ in Eq. [2], so$W^{*}\hat{\mathbf{b}}\left(\mathbf{r}_{k}\right)$ in the other brackets is the appropriate transformation for the coil sensitivities. Separating the single-element spectra into a true signal and a noise term, i.e. $\hat{\mathbf{p}}=\hat{\mathbf{s}}+\hat{\mathbf{n}}$, we then obtain

$\hat{P}\left(\mathbf{r}_{k},\delta_{l}\right)/C=\underbrace{\left[W\hat{\mathbf{s}}\left(\mathbf{r}_{k},\delta_{l}\right)\right]^{T}\left(\frac{W^{*}\hat{\mathbf{b}}\left(\mathbf{r}_{k}\right)}{ \left|W^{*}\hat{\mathbf{b}}\left(\mathbf{r}_{k}\right)\right| }\right)}_{\text{perfect noiseless combined spectrum}}+\underbrace{\left[W\hat{\mathbf{n}}\left(\mathbf{r}_{k},\delta_{l}\right)\right]^{T}\left(\frac{W^{*}\hat{\mathbf{b}}\left(\mathbf{r}_{k}\right)}{ \left|W^{*}\hat{\mathbf{b}}\left(\mathbf{r}_{k}\right)\right| }\right)}_{\text{noise in combined spectrum}}.$ [SI.4]

The first term is the perfect noiseless combined spectrum and the second term is the noise in the combined spectrum. Now, by the definition of the noise whitening matrix $W$, each element of the vector $W\hat{\mathbf{n}}\left(\mathbf{r}_{k},\delta_{l}\right)$ is normally distributed with zero mean and has unit variance. Since, the weighting vector in parentheses (also marked in blue) has unit norm, then the whole right-hand term should have unit variance too.

To test this, we generated in Matlab a $3\times10^{7}$ matrix of multivariate-normally-distributed random numbers with covariance $\hat{\mathbf{\Psi}}=\left(\begin{matrix} 5 & 1+0.6i & 4-0.1i \\ 1-0.6i & 3 & -0.3+0.8i \\ 4+0.1i & -0.3-0.8i & 6 \end{matrix}\right)$.

The measured covariance of this noise, using Eq. [5], was found to be equal to $\hat{\mathbf{\Psi}}$ to within 0.1%.

We then synthesised data by applying Eq. [6] with this noise, Q=1, and.

Applying Roemer's original equation (his Eq. [32]) with the substitution $\hat{\mathbf{R}}\to\hat{\mathbf{\Psi}}$ gave combined data with mean=1.56 and standard deviation (SD)=2.78, which is not right. Meanwhile, applying Eq. [4] with the substitution $\hat{\mathbf{R}}\to\hat{\mathbf{\Psi}}$ gave combined data with mean=0.65 and SD=1.00 as expected.

### Relationship between the WSVD combination method and Adaptive Reconstruction

The WSVD coil sensitivities and maximum likelihood combined spectrum are computed by Eq. [9] and Eq. [20] from the first left-singular vector $U_{\bullet,1}$ and the first singular value $\Sigma_{1,1}$ of S defined in Eq. [8]. Consider the small $N_{i}\times N_{i}$ matrix $S S^{\dagger}$. Now,

$S S^{\dagger}=\left(U\Sigma V^{\dagger}\right)\left(V\Sigma^{\dagger}U^{\dagger}\right)=U\Sigma^{2}U^{\dagger}=U\Delta U^{\dagger}$ [SI.5]

Hence, the first left-singular vector of S is equal to the eigenvector of $S S^{\dagger}$ with largest eigenvalue; and the first singular value of S is equal to

$\Sigma_{1,1}=\sqrt{\Delta_{1,1}}.$ [SI.6]

Substituting Eq. [7] into Eq. [SI.5], we see that

$U^{\dagger}W\bar{S} \bar{S}^{\dagger}W^{\dagger}U=U^{\dagger}S S^{\dagger}U=\Delta=\Sigma^{2}$ [SI.7]

To make the link with Adaptive Reconstruction, we note that our $\bar{S} \bar{S}^{\dagger}$ matrix is equivalent to Walsh's signal covariance matrix $R_{s}$ and our noise covariance matrix $\hat{\mathbf{\Psi}}$ is equivalent to Walsh's noise covariance matrix R_n_.

In Eq. 12–13 of (5), Walsh introduces a transformation matrix P that satisfies

$\text{(a):} P^{H}R_{s}P=D \text{and} (\text{b})\text{:} P^{H}R_{n}P=I.$ [SI.8]

Eq. [SI.7] is equivalent to [SI.6a] iff Walsh's P matrix is equal to our matrix $W^{\dagger}U$. Let us substitute this into [SI.6b] to check it also agrees.

$\left(W^{\dagger}U\right)^{\dagger}\hat{\mathbf{\Psi}}\left(W^{\dagger}U\right)=U^{\dagger}D^{-1/2}\left(X^{\dagger}\hat{\mathbf{\Psi}}X\right)D^{-1/2}U=U^{\dagger}D^{-1/2}DD^{-1/2}U=I$ [SI.9]

So, Walsh's matrix P is indeed equal to our matrix $W^{\dagger}U$.

Substituting this into Walsh's Eq. 14 and applying Eq. [SI.7] shows:

$\begin{matrix} R_{n}^{-1}R_{s}P=\left(\hat{\mathbf{\Psi}}^{-1}\right)\left(\bar{S} \bar{S}^{\dagger}\right)\left(W^{\dagger}U\right) \\ =W^{\dagger}\left(W \bar{S} \bar{S}^{\dagger}W^{\dagger}\right)U \\ =W^{\dagger}U\Delta U^{\dagger}U \\ =\left(W^{\dagger}U\right)\left(\Delta\right)=PD. \end{matrix}$ [SI.10]

So, just as in the case of Adaptive Reconstruction, $P=W^{\dagger}U$ and $D=\Delta=\Sigma^{2}$ are eigenvectors and eigenvalues of the matrix $R_{n}^{-1}R_{s}$. Hence, the **WSVD** algorithm maximum likelihood spectrum formula in Eq. [20] is mathematically equivalent to application of Adaptive Reconstruction's "optimal processor", that is the eigenvector corresponding to the maximum eigenvalue of the matrix $R_{n}^{-1}R_{s}$.

Nevertheless, the derivation and physical interpretation of this result is different for the **WSVD** combination method and for Adaptive Reconstruction. Adaptive Reconstruction is derived using the theory of stochastic processes, whereas the **WSVD** combination method is derived using Bayes' theorem and properties of the multivariate normal distribution. Adaptive Reconstruction is applied to e.g. 16x16 sets of voxels within an image (implicitly assuming that B_1_^–^ is constant for each element in that small area), whereas the **WSVD** combination method is applied to all the points in the single-element spectra (or FIDs) from a single voxel.

### Time-domain variant of the WSVD combination algorithm

We previously observed that the **WSVD** combination algorithm's derivation applies equally to the combination of single-element spectra or single-element FIDs (3). We prove here that, providing the same noise covariance matrix is employed in both cases (e.g. from a pre-scan), the resulting coil sensitivities are identical after a time- or frequency-domain calculation and hence the resulting maximum likelihood combined FID and spectrum form a Fourier pair. This means that online reconstruction code can use data in whichever domain is most efficient.

The discrete Plancherel theorem (6) states that for two vectors **x** and **y** with discrete Fourier transforms **X** and **Y** respectively

$\sum_{n=0}^{N-1} x_{n}y_{n}^{*}=\frac{1}{N}\sum_{k=0}^{N-1} X_{k}Y_{k}^{*}.$ [SI.11]

In terms of the raw single-element FIDs $\bar{s}$ or spectra $\bar{S}$, applying Eq. [SI.11] gives

$\left(\bar{S} \bar{S}^{\dagger}\right)_{ik}=\sum_{j=0}^{N_{s}-1} \bar{S}_{ij} \bar{S}_{kj}^{*}=N_{s}\sum_{j=0}^{N_{s}-1} \bar{s}_{ij} \bar{s}_{kj}^{*}=N_{s}\left(\bar{s} \bar{s}^{\dagger}\right)_{ik}$ [SI.12]

Hence, converting to whitened channels via Eq. [7], we write

$SS^{\dagger}=W\left(\bar{S} \bar{S}^{\dagger}\right)W^{\dagger}=W\left(N_{s} \bar{s} \bar{s}^{\dagger}\right)W^{\dagger}=N_{s}s s^{\dagger}$ [SI.13]

Since the number of samples $N_{s}$ is a scalar, the unitary matrix of left-singular vectors U computed from whitened frequency-domain ($SS^{\dagger}$) or time-domain ($ss^{\dagger}$) data via Eq. [SI.5] is therefore identical, apart from an arbitrary overall phase. Hence, the same linear combination of single-element spectra or FIDs is produced whether we perform **WSVD** processing in the time- or frequency-domain.

### Per-element gain/phase calibration from low SNR data: the DWALS algorithm

In Appendix 1, we outlined a procedure to calibrate a per-element gain/phase coefficient from high SNR phantom data. It is also possible in theory to compute this calibration directly from low SNR data in-vivo as follows. By analogy with Eq. [13], the whitened signals from channel *i,* at voxel *k,* and chemical shift $\delta_{l}$ satisfy

$S_{i}\left(\mathbf{r}_{k},\delta_{l}\right)\approx\beta_{i}^{*}B_{1 \text{,} \text{BS,} i}^{-}\left(\mathbf{r}_{k}\right)^{*}\hat{M}_{xy}\left(\mathbf{r}_{k},\delta_{l}\right) + \xi_{i}$ [SI.14]

where $\beta_{i}$ is again the element's scaling coefficient, $B_{1 \text{,} \text{BS,} i}^{-}\left(\mathbf{r}_{k}\right)$ is the Biot-Savart sensitivity, $\hat{M}_{xy}\left(\mathbf{r}_{k}\right)$ is the sample magnetisation and $\xi_{i}$ represents noise drawn from independent, normally-distributed distributions with unit variance. Introducing a flattened index K, we write

$S_{iK}=\beta_{i}^{*}B_{1,BS\text{,} iK}^{-*}m_{K}=W_{iK}\beta_{i}^{*}m_{K}+\xi_{i} \text{where} W_{iK}=B_{1 \text{,} BS, iK}^{-*}.$ [SI.15]

Solving Eq. [SI.15] for *several voxels* simultaneously imposes the *shape* of the Biot-Savart fields as prior knowledge but allows their overall magnitude and phase to vary freely.

Eq. [SI.15] is known as a *weighted* rank-1 matrix decomposition problem (7-12), which can be solved using the alternating least squares (ALS) algorithm (10,12). The key observation is that the optimal solution of Eq. [SI.15] minimises

$\rho=\sum_{i\text{,}K} \left|S_{iK}-W_{iK}\beta_{i}^{*}m_{K}\right|^{2}.$ [SI.16]

This can be rearranged into one of two linear least squares problems if either $\beta_{i}$ or $m_{k}$ are known:

$\rho=\sum_{a, b} \left|\left(\hat{W}_{\beta}\right)_{ab}m_{b}-\text{vec}\left(S\right)_{a}\right|^{2}=\sum_{a, b} \left|\left(\hat{W}_{m}\right)_{ab}\beta_{b}^{*}-\text{vec}\left(S\right)_{a}\right|^{2}$ [SI.17]

where the banded sparse $N_{i}N_{k}\times N_{i}$ matrix $\hat{W}_{\beta}$ incorporates $W_{iK}$ and $\beta_{i}^{*}$; $\hat{W}_{m}$ incorporates $W_{iK}$ and $m_{k}$; and *vec* flattens a matrix into a column vector. The ALS algorithm starts from a random initial $\beta_{i},$ solves for $m_{k}$, then solves for $\beta_{i}$, repeating until the solution converges. The final $\beta_{i}$ can then be used in Eq. [17] to compute the calibrated sensitivities for the Roemer combination method via Eq. [4].

If only one voxel is used, these decorrelated weighted alternating least squares (“**DWALS**”) coil sensitivities are identical to the maximum likelihood sensitivities computed by the **WSVD** combination algorithm in that voxel. This was confirmed experimentally.

We mention the **DWALS** combination method for completeness, but we do not show results in the main manuscript because we found the **DWALS** combination method to perform no better in-vivo than the simpler "**Roemer (BS B_1_^-^ phased)**" phantom pre-calibration combination method. This implies that the problem with the Biot-Savart B_1_^–^ fields is that they are qualitatively the wrong shape, rather than being mis-phased or mis-scaled.

## Supporting References

1. Brown R, Wang Y, Spincemaille P, Lee RF. On the noise correlation matrix for multiple radio frequency coils. Magn Reson Med 2007;58(2):218-224.

2. Ohliger MA, Sodickson DK. An introduction to coil array design for parallel MRI. NMR Biomed 2006;19(3):300-315.

3. Rodgers CT, Robson MD. Receive Array Magnetic Resonance Spectroscopy: Whitened Singular Value Decomposition (WSVD) Gives Optimal Bayesian Solution. Magn Reson Med 2010;63(4):881-891.

4. Arfken GB, Weber H-J. Mathematical methods for physicists. Amsterdam ; London: Elsevier Academic; 2005.

5. Walsh DO, Gmitro AF, Marcellin MW. Adaptive reconstruction of phased array MR imagery. Magn Reson Med 2000;43(5):682-690.

6. Bracewell RN. The Fourier transform and its applications. New York ; London: McGraw-Hill; 1986.

7. Srebro N, Jaakkola T. Weighted low-rank approximations. Twentieth International Conference on Machine Learning (ICML-2003). Volume 3. Washington DC; 2003. p 720-727.

8. Markovsky I. Algorithms and literate programs for weighted low-rank approximation with missing data. Approximation Algorithms for Complex Systems; DOI: Springer; 2011. p 255-273.

9. Lu WS, Pei SC, Wang PH. Weighted low-rank approximation of general complex matrices and its application in the design of 2-D digital filters. Circuits and Systems I: Fundamental Theory and Applications, IEEE Transactions on 1997;44(7):650-655.

10. Okatani T, Deguchi K. On the Wiberg Algorithm for Matrix Factorization in the Presence of Missing Components. Int J Comput Vision 2007;72(3):329-337.

11. Zachariah D, Sundin M, Jansson M, Chatterjee S. Alternating Least-Squares for Low-Rank Matrix Reconstruction. Ieee Signal Proc Let 2012;19(4):231-234.

12. Eriksson A, van den Hengel A. Efficient Computation of Robust Weighted Low-Rank Matrix Approximations Using the L-1 Norm. Ieee T Pattern Anal 2012;34(9):1681-1690.
